# The lncRNA MACC1-AS1 promotes gastric cancer cell metabolic plasticity via AMPK/Lin28 mediated mRNA stability of MACC1

**DOI:** 10.1186/s12943-018-0820-2

**Published:** 2018-03-06

**Authors:** Yang Zhao, Yajing Liu, Li Lin, Qiong Huang, Wanming He, Shuyi Zhang, Shumin Dong, Zhaowei Wen, Jinjun Rao, Wangjun Liao, Min Shi

**Affiliations:** 1 Department of Oncology, Nanfang Hospital, Southern Medical University, Guangzhou, China; 2 Key laboratory of new drug screening of Guangdong province, School of Pharmaceutical Sciences, Southern Medical University, Guangzhou, China

**Keywords:** MACC1-AS1, MACC1, Long non-coding RNA, Gastric cancer, Metabolic plasticity, mRNA stability

## Abstract

**Background:**

Metabolic plasticity has been increasingly thought to be a determinant of tumor growth and metastasis. MACC1, a transcriptional regulator of MET, was recognized as an oncogene in gastric cancer (GC); however, its transcriptional or post-translational regulation was not clear. We previously reported the metabolic role of MACC1 in glycolysis to promote GC progression. MACC1-AS1 is the antisense lncRNA of MACC1, yet its function was previously unknown.

**Methods:**

We profiled and analyzed the expression of MACC1-AS1 utilizing the TCGA database as well as in situ hybridization using 123 pairs of GC tissues and matched adjacent normal gastric mucosa tissues (ANTs). The biological role of MACC1-AS1 in cell growth and metastasis was determined by performing in vitro and in vivo functional experiments. Glycolysis and antioxidant capabilities were assayed to examine its metabolic function. Further, the specific regulatory effect of MACC1-AS1 on MACC1 was explored transcriptionally and post-transcriptionally.

**Results:**

MACC1-AS1 was shown to be expressed significantly higher in GC tissues than in ANTs, which predicted poor prognosis in GC patients. MACC1-AS1 promoted GC cell proliferation and inhibited cell apoptosis under metabolic stress. Mechanistically, MACC1-AS1 stabilized MACC1 mRNA and post-transcriptionally augmented MACC1 expression. Further, MACC1-AS1 was shown to mediate metabolic plasticity through MACC1 upregulation and subsequent enhanced glycolysis and anti-oxidative capabilities, and this was suggested to be coordinated by the AMPK/Lin28 pathway.

**Conclusions:**

Elevated expression of MACC1-AS1 in gastric cancer tissues is linked to poor prognosis and promotes malignant phenotype upon cancer cells. MACC1-AS1 is elevated under metabolic stress and facilitates metabolic plasticity by promoting MACC1 expression through mRNA stabilization. Our study implicates lncRNA MACC1-AS1 as a valuable biomarker for GC diagnosis and prognosis.

**Electronic supplementary material:**

The online version of this article (10.1186/s12943-018-0820-2) contains supplementary material, which is available to authorized users.

## Background

Gastric cancer (GC) is the fourth most common cancer and the second leading cause of cancer-related death worldwide [1]. Effective treatment is lagging for this disease, and the underlying mechanism is still not generally understood. Metabolic plasticity is considered one of the defining hallmarks of cancer, suggesting that this could be a potential target for GC diagnosis and therapeutics [2].

MACC1 is a transcriptional regulator of MET, and has emerged as a master oncogene that is upregulated in a variety of tumors, promoting proliferation, invasion, and chemotherapy resistance [3]. Our previous study demonstrated that MACC1 expression is upregulated and promotes glycolysis under metabolic stress, a state characterized by nutrient deprivation and often occurring in mature tumor tissues due to abnormal vascularization [4, 5]. However, the specific regulation of MACC1 under metabolic stress remains poorly understood.

LncRNAs have emerged as a concept in biology, playing integral roles in the control of a wide variety of cellular functions through transcriptional and post-transcriptional regulation of gene activity [6]. MACC1-AS1 is the cognate antisense lncRNA of MACC1, and to date, its function and molecular mechanism have not been investigated. Generally, antisense lncRNA exerts regulatory effects on the expression of its sense counterpart. This relationship is also supported through the analysis of MACC1-AS1 and MACC1 expression using the TCGA database and tumor specimens from patients. Based on this, we suspected that MACC1-AS1 might regulate MACC1 expression, and the potential mechanism was further explored in this study.

Herein, we provide new evidence that MACC1-AS1 lncRNA is highly expressed in GC, which was found to be correlated with advanced clinical stage and poor prognosis. MACC1-AS1 potentiated metabolic plasticity through enhanced glycolysis and antioxidant activity under metabolic stress, which was found to be mediated through post-translational regulation of MACC1 mRNA stability.

## Methods

### Reagents

AMPK inhibitor dorsomorphin was purchased from Selleck (Houston, TX, USA); 2-Deoxy-D-glucose (2-DG), Cycloheximide (CHX), Dactinomycin D from MedChem Express (Princeton, MA, USA); 3-(4,5- dimethylthiazol-2-yl)-2,5-diphenyl tetrazolium bromide(MTT) from Biosharp (China); 2-NBDG (2-(N-(7-Nitrobenz-2-oxa-1,3-diazol-4-yl)Amino)-2 Deoxyglucose) and 4′,6-diamidino-2-phenylindole(DAPI) from Invitrogen; H_2_O_2_, N-acetyl-L-cysteine (NAC) from Sigma-Aldrich; Other reagents were mentioned for below procedure.

### Clinical samples

A total of 123 formalin-fixed and paraffin-embedded (FFPE) GC tissue samples from patients who had underwent an operation in Nanfang Hospital (Guangzhou, China) between January 2006 and December 2010 were included in the cohort. All patients were staged based on the criteria of the 7th Edition of the AJCC Cancer Staging Manual: Stomach (2010) [7]. No recruited patients received any preoperative treatment. For stage I–III patients, prognosis was assessed based on disease-free survival (DFS), whereas for stage IV patients were analyzed based on overall survival (OS). All patients provided written informed consent before the study, which was approved by the Nanfang Hospital Ethics Review Committee (Guangzhou, China).

### Immunohistochemical (IHC) and in situ hybridization (ISH) staining

Briefly, IHC was applied to detect the expression of MACC1 (Abcam, San Francisco, CA), whereas ISH was utilized to detect the presence of MACC1-AS1 using a double digoxigenin (DIG)-labeled mercury locked nucleic acid probe (5’DigN/TCAATGCAGATCTAATACTCCT/3’Dig_N) (miRCURY LNA™,Exiqon, Vedbaek, Denmark). Staining score was assessed by two independent reviewers as: 0 = no staining, 1 = weak staining, 2 = moderate staining, 3 = strong staining. Tumor cells in five fields were selected randomly and scored based on the percentages of positive stained cells (1 = 0-25%, 2 = 26-50%, 3 = 51-75%, 4 = 76-100%). The final scores were computed by multiplying the intensity score and the percentage score of positive cells. According to scores, samples were divided into three groups, the negative expression (score 0-2), low expression (score 3-7) and high expression group (score 8-12). In the survival analysis, negative and low scores group were defined as MACC1-AS1 negative group (−), while high expression group were defined as MACC1-AS1 positive group (+).

### Cell culture and treatment

Five human GC cell lines (AGS, BGC803, BGC823, MKN45, SGC7901) and immortalized human gastric epithelial cell line (GES-1) were obtained from Foleibao Biotechnology Development Co. (Shanghai, China). All cell lines were cultured in RPMI 1640 medium supplemented with 10% fetal bovine serum (HyClone, Logan, UT, USA) at 37 °C under 5% CO_2_. Glucose deprivation was used to induce metabolic stress using glucose-free 1640 medium (GIBCO, USA). MKN45 and BGC803 cells stably transfected with pLenti-EF1a-Luc-F2A-puro-CMV-MACC1-AS1 constructs or pLKD-U6-shRNA-EF1a-LUC-F2A-Puro-MACC1 (Obio Technology, shanghai, China) were grown in 1 μg/ml puromycin (Invitrogen).

### Vector construction

The cDNA of MACC1-AS1 was PCR-amplified and subcloned into pcDNA 3.1 (+) vector (Invitrogen). The pcDNA3.1-antisense-MACC1-AS1 was constructed by subcloning the antisense MACC1-AS1 sequence into the pcDNA3.1 (−) vector (Invitrogen). The putative MACC1 promoter was PCR-amplified and cloned into the pGL3-control vector (Promega) to construct the pGL3-MACC1-3′UTR vector. All products were validated by DNA sequencing.

### Small interfering RNA transfection

Cells were plated and cultured in growth media until cell density reached to 30-40% prior to siRNA transfection using Lipofectamine 2000 (Invitrogen). siRNA sequences are as following: AMPK (5’-CTATGCTGCACCAGAAGTA-3′), Lin 28 (5′- GAAGAAATCCACAGCCCTA-3′), and synthetic sequence-scrambled siRNA was used as a negative control.

### Biotin pull-down assays and mass spectrometry

MACC1-AS1 and its antisense control sequence were in vitro transcribed from pGEM-T plasmid and biotin-labeled with Biotin RNA Labeling Mix (Roche) and T7 RNA polymerase (Roche). 1 mg of whole-cell lysates from MKN45 cells were incubated with purified biotinylated transcripts for 1 h at room temperature. Complexes were isolated with streptavidin agarose beads (Invitrogen). The RNA present in the pull-down material was detected by qRT-PCR analysis, and retrieved proteins were detected by western blot analysis or resolved in gradient gel electrophoresis followed by mass spectrometry (MS) identification (TripleTOF 5600 LCMS, AB SCIEX).

### Next-generation RNA sequencing (RNA-seq)

RNA-seq of MACC1-AS1-overexpressing or vector-transfected MKN45 cells were carried out using the platform BGIseq-500 (Shenzhen, China). Gene set enrichment analysis (GSEA) was carried out using GSEA v3.0.

### RNA isolation and qPCR analysis

Total RNA of cells were extracted by TRIZOL reagent (Invitrogen), the cDNA was synthesized by using the first strand cDNA synthesis kit (Takara Shuzo, Kyoto, Japan). Quantitative PCR was applied using the SYBR Green dye (Takara, Japan) on LightCycler 480 system (Roche, Penzberg, Germany). The primer sequences were listed in the Additional file 1: Table S7. The relative expression was normalized to snRNA U6 or β-actin by 2^-ΔΔCt^ method.

### Western blotting

Cells were harvested and lysed on ice in RIPA buffer (50 mM Tris, 100 mM NaCl, pH 8.0, 10 mM EDTA, pH 7.0, 0.4% *v*/v Triton X-100, 10 mM nicotinamide) containing protease and phosphatase inhibitors (Beyotime, Shanghai, China). Lysates were centrifuged at 15,000 g at 4 °C for 15 min. Proteins were resolved using SDS–polyacrylamide gel electrophoresis and transferred onto PVDF membrane. Membranes were blocked in 5% milk, incubated with primary antibody at recommended concentration in TBST with 5% BSA overnight at 4 °C, then incubated with fluorescent-tagged secondary antibody at a concentration of 1:15,000 and read using a LI-COR Odyssey infrared imaging system. The primary antibody used were: anti-MACC1, anti-GLUT1, anti-HK2, anti-Ecadherin, anti-Ki67 (Abcam, Cambridge, MA); anti-pAMPK (Thr 172), anti-GAPDH (ImmunoWay, New York, DE, USA); anti-LDHA, anti-Lin28, anti-β-actin and anti-Histone H3 (Proteintech Group, Chicago, IL, USA); anti-caspase 3, anti-bax, anti-Ncadherin (Abclonal, Cambridge, MA, USA); anti-8-OHdG (Japan Institute for the Control of Aging, Shizuoka, Japan). β-actin or Histone H3 was used as a loading control.

### Luciferase assays

Cells were transfected with the pGL3-MACC1-3′UTR vector and the renilla luciferase plasmid (pRL-TK). Then, the cells were harvested after 36 h for firefly/renilla luciferase assays using the Dual-Luciferase Reporter Assay System (Promega). Luciferase activities were normalized to the pRL-TK plasmid.

### FISH and Immunofluorescence analysis

To detect MACC1-AS1 expression in cells, Cy3-labeled probes was synthesized for fluorescence in situ hybridization (FISH). Cells were rinsed in PBS and fixed in paraformaldehyde (Solarbio, China) for 15 min at room temperature. Next, cells were permeabilized in 0.5% Triton X-100 on ice for 10 min, and washed in PBS for 3 times. Hybridization was carried out using Fluorescent in Situ Hybridization Kit (RIBO Bio, Guangzhou, PR China) in a moist dark chamber at 37 °C for 12 h according to the protocol. After serial saline-sodium citrate (SSC) buffer washing, MACC1-AS1 was detected under confocal laser microscope (Olympus Optical, Tokyo, Japan). For co-localization study, cells were fixed again in paraformaldehyde and subjected to immunofluorescence, 4′, 6-diamidino-2-phenylindole (DAPI) was used to stain the nucleus.

### Cell nuclear and cytoplasmic RNA and protein isolation

For cytoplasmic fraction, cells were washed with ice-cold PBS for three times, then the PARIS Kit (Life Technologies, Carlsbad, CA, USA) was applied. Briefly, cells were lysed by cell fractionation buffer and incubated on ice for 10 min before centrifuging at 4 °C, 500 g for 5 min. The supernatant was collected as cytoplasmic fraction while the remaining nuclear pellets were lysed again by cell disruption buffer as nuclear fraction. Both the cytoplasmic and nuclear fraction were proceeded to isolate the RNA and protein by following binding and washing procedure according to the protocol. The nuclear and cytoplasmic RNA and protein was further analyzed by western blotting or qPCR.

### 2-NBDG uptake assay

After 12 h culture in conditions of either normal or glucose-free medium, recipient cells were labelled with 100 μM 2-NBDG diluted in glucose-free medium, and incubated for 30 min at 37 °C. Fluorescence quantification was carried out using flow cytometry at emission 465/excitation 540 nm.

### Metabolite, ROS and enzyme activities

MACC1-AS1-overexpressing or vector-transfected MKN45 cells were seeded on 6-well plates at a density of 2 × 10^5^ cells per well. 48 h later, the culture medium was changed to glucose-free medium and cultured for another 12 h. Cell supernatant was collected to measure lactate concentration (Nanjing Jiancheng Bioengineering Institute, Nanjing, China), while the cell pellets to be lysed and measured ATP (Beyotime, Haimen, China) and the activity of HK and LDH (Comin, Suzhou, China). ROS was measured by fluorescent 2′, 7′-dichlorofluorescin diacetate (DCF-DA) as described by manufacturer’s protocol of commercial kit (Nanjing Jiancheng, China).

### NADP+/NADPH and GSH/GSSG assays

After 12 h of glucose deprivation, the recipient cells were collected and analyzed using NADP+/NADPH quantification kit (Biovision K347, CA, USA) and GSH/GSSG quantification kit (Biovision, USA) according to the manufacturer’s instructions.

### Cell apoptosis, viability and cell cycle analysis

Cell apoptosis was measured with Annexin/PI kit (Key Gen Bio TECH, Nanjing, China) by flow cytometry analysis (BD Biosciences, San Jose, CA, USA). Cell cycle was measured with cell cycle detection kit (Key Gen, China). Cell viability was measured by MTT assays and EdU assays (RIBO, China) according to the protocol.

### Colony formation assay

The MACC1-AS1-overexpressing or vector-transfected MKN45 cells were seeded in 6-well plates at 1× 10^3^ per well and cultured for 2 days. The medium was changed to culture in low concentration of glucose medium for another 2 weeks. After that, the cells were washed twice with PBS, fixed with paraformaldehyde and stained with 0.5% crystal violet. After washing again, the plates were photographed under a microscope.

### Cell migration/ invasion assay

For transwell migration assays, The MACC1-AS1-overexpressing or vector-transfected MKN45 cells were plated in the top chamber with the non-coated membrane (BD Biosciences, San Jose, CA, USA). For invasion assays, matrigel (BD biosciences) was polymerized in transwell inserts for 2 h at 37 °C. Transfected cells were plated in the top chamber in medium without serum. In both assays, the lower chamber filled with 10% fetal bovine serum. Cells were incubated for 24 h and the cells that did not migrate or invade through the pores were removed by a cotton swab. Cells on the lower surface of the membrane were stained with crystal violet and photographed.

### In vivo metastasis assays

Ten 4-week-old BALB/c nude mice were injected with MACC1-AS1-overexpressing or vector-transfected MKN45 cells in each group. Briefly, 5*10^5^ cells were injected intravenously through the tail vein. Six weeks after injection, the mice were sacrificed and examined by H&E and IHC staining.

### Bioinformatics analysis

The expression of MACC1-AS1 was profiled based on normalized RNA-seq expression data sets of the stomach adenocarcinoma (STAD) cohort study from the Broad TCGA GDAC website (http://gdac.broadinstitute.org/). Up until March 2017, STAD had collected data on 32 normal and 375 cancerous tissues. We defined significant and differential expression of a gene, between tumors and normal samples, based on two conditions: false discovery rate adjusted *P*-value < 0.01 and fold change > 2 [8]. The Bioconductor package edgeR was used to analyze *P* values and fold-changes using an R statistical environment (version 3.3.2).

### Statistical analysis

Statistical analysis was performed using SPSS version 20 software (SPSS, Chicago, IL, USA) or R software (version 3.3.2). Differences between experimental groups were assessed by Student’s t-test or one-way analysis of variance. Chi-squared test and Mann-Whitney test were applied where appropriate to analyse the association between MACC1-AS1 expression and clinicopathological parameters. Spearman correlation were used to analyse the R^2^ between MACC1-AS1 and MACC1 for the serial staining. The log-rank test was performed to compare the survival curves of individual groups. Cox regression was used for univariate and multivariate analysis. The reported results included hazard ratios (HR) and 95% confidence intervals (CI). All values were expressed as mean ± SD, and statistical significance was noted as *P* < 0.05.

## Results

### MACC1-AS1 is upregulated in GC tissues and predicts poor prognosis

MACC1-AS1 is located on the anti-sense strand of the *MACC1* gene; further bioinformatics analysis failed to predict coding probability of more than 0.05 (Additional file 2: Figure S1A-C), supporting the fact that MACC1-AS1 is a lncRNA without protein coding potential.

**Fig. 1:**
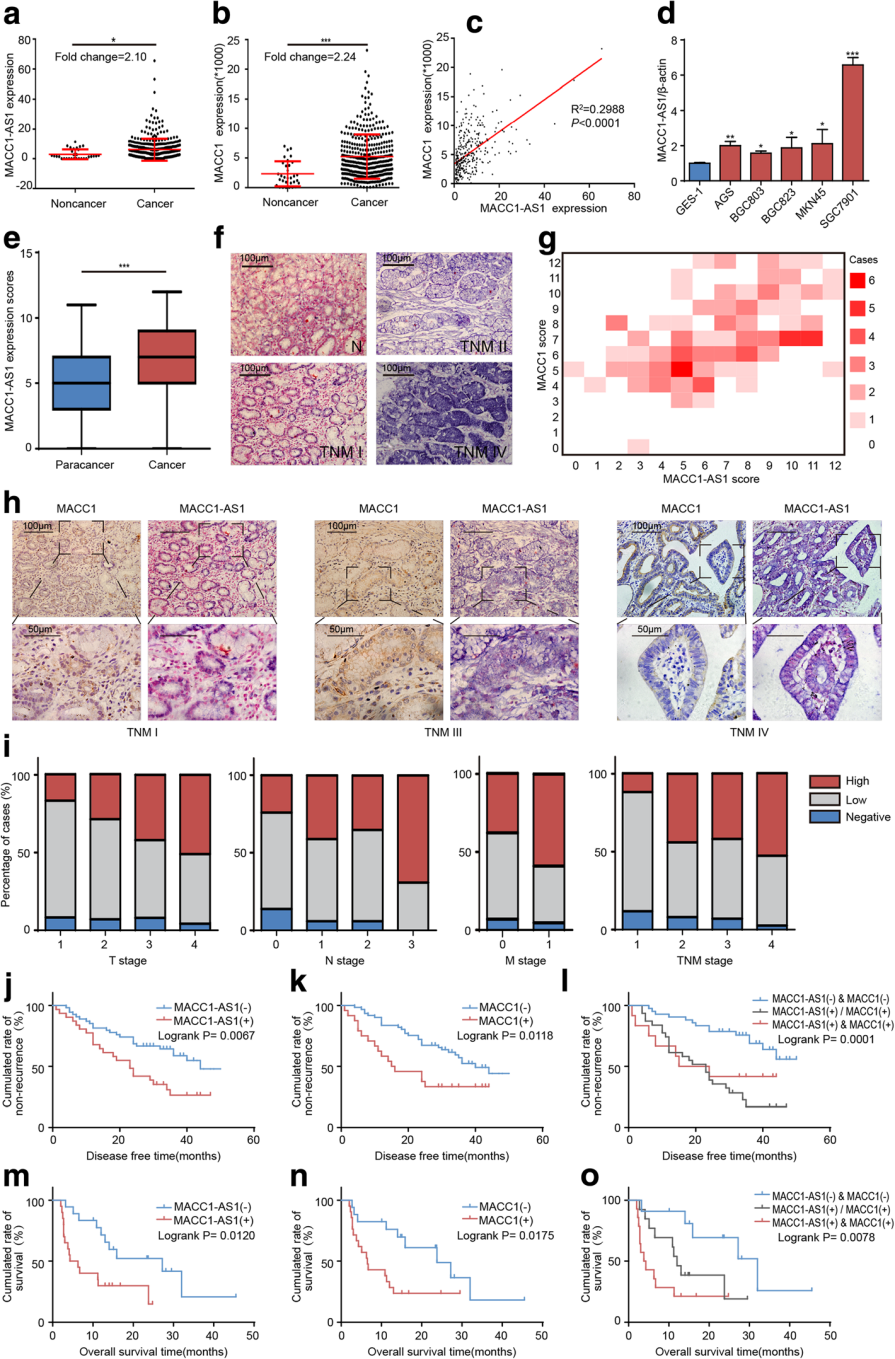
MACC1-AS1 is upregulated in gastric cancer (GC) tissues and predicts poor prognosis. **a**–**b** RNA-seq data from the TCGA database showed that levels of MACC1-AS1 and MACC1 are elevated in GC tissues, compared to that in normal counterparts; *P < 0.05, ***P < 0.001. **c** Correlation analysis showing that MACC1-AS1 is positively correlated with MACC1 expression. **d** qPCR results showing baseline mRNA levels of MACC1-AS1 in GES1 and five GC cell lines; *P < 0.05, **P < 0.01, ***P < 0.001. **e** In situ hybridization of MACC1-AS1 showed that MACC1-AS is upregulated in GC compared to expression in adjacent normal gastric mucosa tissues (ANTs); ***P < 0.001. **f** Representative images of MACC1-AS1 expression in GC tissues of different TNM stages. **g** MACC1-AS1 expression was positively correlated with MACC1 expression in paired non-cancerous and cancer tissues. The horizontal axis of the heatmap shows MACC1-AS1 scores, whereas the vertical axis shows MACC1 scores. The crossing grid indicates the number of cases (based on different colors). **h** Representative images of successive IHC/ISH staining of MACC1 and MACC1-AS1 expression in GC tumor tissues of different TNM stage. **i** Frequency of cases of negative, low, and high MACC1-AS1 expression in GC samples categorized by TNM stage. **j**–**o** Elevated expression of MACC1-AS1 and MACC1 was found to be associated with reduced disease-free survival (J-L) in stage I–III GC patients and overall survival (**m**-**o**) in stage IV patients

To investigate differential gene expression in GC, we firstly analyzed the stomach adenocarcinoma (STAD) cohort study containing 32 normal and 375 GC tissues from the TCGA database. The genes significantly upregulated by 2-fold in tumor tissues compared to normal counterparts were screened out (Additional file 3: Table S1). Then the correlation coefficient was calculated and the top 500 ranked genes linearly correlated to MACC1 expression were selected (Additional file 4: Table S2). The intersection contained 38 genes and both MACC1-AS1 and MACC1 were included, suggesting that expression of both is upregulated in GC, and exhibits a positive linear correlation (R^2^ = 0.2988, *P* < 0.001) (Fig. 1a–c and Additional file 2: Figure S1D). In addition, this correlation between MACC1-AS1 and MACC1 was further validated in GC and breast cancer cell lines and compared to that in an immortalized non-cancer cell line (Fig. 1d and Additional file 5: Figure S2A–B). Next, we performed in situ hybridization to measure MACC1-AS1 expression in 123 pairs of GC samples and matched adjacent normal gastric mucosa tissues (ANTs). The clinicopathological characteristics of these patients are summarized in Table 1. Generally, MACC1-AS1 was expressed at a higher level in GC tissues compared to that in adjacent ANTs (Fig. 1e). MACC1-AS1 expression increased with clinical stage (Fig. 1f), and displayed a positive correlation with MACC1 expression based on successive immunohistochemistry (IHC) staining (Fig. 1g-h).

**Table 1:** Correlation between MACC1-AS1, MACC1 and clinicopathological parameters

*n* (%)		MACC1-AS1			MACC1		
		Low and negative (*n*)	High (*n*)	*P*	Low and Negative (n)	High (*n*)	*P*
Age (years)				0.752			0.803
≥55	72(58.5%)	43	29		45	27	
<55	51(41.5%)	29	22		33	18	
Sex				0.351			
Male	76(61.8%)	42	34		45	31	0.22
Female	47(38.2%)	30	17		33	14	
TNM stage				0.022*			0.002**
I	17(13.8%)	15	2		14	3	
II	25(20.3%)	14	11		19	6	
III	43(35.0%)	25	18		28	15	
IV	38(30.9%)	18	20		17	21	
Tumor invasion				0.024*			0.135
T1	12(9.8%)	10	2		9	3	
T2	14(11.4%)	10	4		11	3	
T3	50(40.7%)	29	21		31	19	
T4	47(38.2%)	23	24		27	20	
Lymph node metastasis				0.004**			0.034*
N0	29(23.6%)	22	7		13	13	
N1	34(27.7%)	20	14		20	14	
N2	34(27.7%)	22	12		23	11	
N3	26(21.1%)	8	18		22	7	
Distant metastasis				0.065			0.055
M0	101(82.1%)	63	38		68	33	
M1	22(17.9%)	9	13		10	12	
Tumor differentiation				0.963			0.923
Well	6(4.9%)	5	1		5	1	
Moderate	20(16.3%)	10	10		11	9	
Poor	97(78.9%)	57	40		62	35	
Mortality				0.213			0.138
Survive	13(34.2%)	8	5		8	5	
die	25(65.8%)	10	15		9	16	
Recurrence				0.012*			0.114
No	40(47.1%)	31	9		32	8	
Yes	45(52.9%)	23	22		29	16	

*P<0.05; **P<0.01

After demonstrating that MACC1-AS1 is upregulated in GC and is co-expressed with MACC1, we analyzed MACC1-AS1 expression as it relates to different clinicopathological factors. As shown in Fig. 1i, when compared to TNM stage, MACC1-AS1 expression was more frequently observed in patients with more advanced T stage (*P* < 0.05), N stage (*P* < 0.05), and overall TNM stage (*P* < 0.05). Kaplan–Meier analysis showed that higher MACC1-AS1 or MACC1 expression was associated with reduced DFS (stage I–III GC patients) and OS (stage IV GC patients) compared to that in the corresponding low and negative expression groups (Fig. 1j–o). Univariate and multivariate Cox proportional hazards analysis showed that MACC1-AS1, MACC1, and TNM stage were independent prognostic factors in GC patients (Table 2). Taken together, these results indicated that MACC1-AS1 is a predictor of poor survival for GC.

**Table 2:** Univariate and multivariate Cox regression analysis for mortality in stage III and IV GC patients

Variables	Univariate analysis		Multivariate analysis	
	HR (95%CI)	*P*	HR (95%CI)	*P*
I-III stage				
Age (≥55 vs. < 55)	0.832(0.447-1.548)	0.562	0.808(0.416-1.572)	0.53
Sex (Male vs. Female)	0.842(0.453-1.566)	0.587	1.166(0.596-2.284)	0.653
TNM stage (I-III)	2.157(1.359-3.423)	0.001**	2.208(1.368-3.562)	0.001**
MACC1 expression(high vs. low and negative)	2.144(1.160-3.963)	0.015*	2.004(1.054-3.809)	0.034*
MACC1-AS1 expression (high vs. low and negative)	2.194(1.218-3.952)	0.009**	1.913(1.041-3.516)	0.037*
IV stage				
Age (≥55 vs. < 55)	1.121(0.493-2.546)	0.786	1.722(0.675-4.395)	0.255
Sex (Male vs. Female)	0.971(0.423-2.226)	0.944	2.162(0.726-6.445)	0.166
MACC1 expression((high vs. low and negative)	2.751(1.155-6.554)	0.022*	2.889(1.122-7.443)	0.028*
MACC1-AS1 expression (high vs. low and negative)	2.572(1.162-5.692)	0.020*	2.509(1.025-6.143)	0.044*

*Abbreviations: HR* hazard ratio, *CI* confidence interval, *P<0.05, **P<0.01

### MACC1-AS1 is induced under metabolic stress and promotes GC progression

**Fig. 2:**
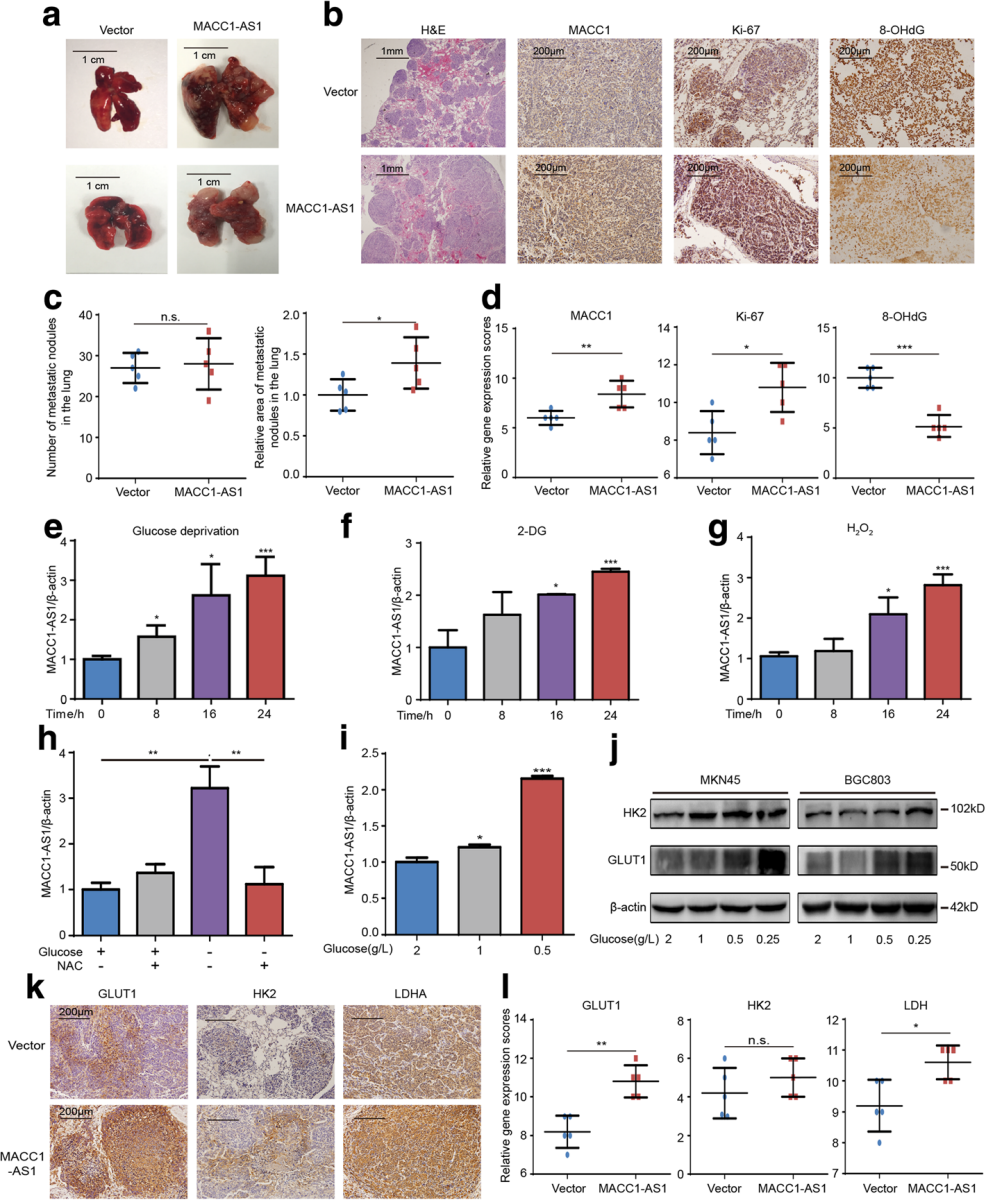
MACC1-AS1 is induced under metabolic stress and promotes GC progression. **a**-**b** MACC1-AS1-overexpressing or vector-transfected MKN45 cells were injected into the caudal vein of nude mice (*n* = 5, 1 × 10^6^ per mouse). Gross lung metastatic tumors (**a**) and H&E stained sections of lungs showed that overexpression of MACC1-AS1 promoted the metastasis of GC. Immunohistochemical staining showed that the MACC1-AS1 overexpression group exhibited higher MACC1 expression with higher rates of proliferation, as measured by Ki-67 staining, and lower numbers of oxidative lesions, based on 8-OHdG staining (**b**). **c** Quantitative analysis of lung metastatic nodules showed that the number of nodules were not significantly different, while MACC1-AS1 overexpression group exhibited larger relative area of nodules of the lung. **d** Quantitative analysis of IHC staining showing MACC1-AS1 overexpression group exhibited higher expression of MACC1, Ki-67 but lower expression of 8-OHdG. **e**-**g** qPCR results showing that MACC1-AS1 was increased after glucose deprivation (**e**), 2-DG (10 mM) (**f**) or H_2_O_2_ (100 μM) (**g**) treatment. *P < 0.05, ***P < 0.001. **h** qPCR results showing that glucose deprivation-derived MACC1-AS1 upregulation was reversed by NAC treatment (8 mM). **P < 0.01. **i** qPCR results showing that MACC1-AS1 was upregulated under culture conditions of sustained low glucose for 1 month (1, 0.5 g/L) compared to normal glucose (2 g/L). *P < 0.05, ***P < 0.001. **j** Western blotting results showing that GLUT1 and HK2 were increased under culture conditions of sustained low glucose for 1 month. **k**-**l** Representative staining and quantitative analysis of lung metastatic nodules showing MACC1-AS1 overexpression group exhibited higher expression of GLUT1, HK2 and LDH

To evaluate whether MACC1-AS1 is functionally involved in GC progression, we overexpressed MACC1-AS1 in both transient plasmid and stable lentivirus transfected ways, the overexpression efficiency was examined and the established stable cell lines were used for latter functional studies (Additional file 6: Figure S3A–B). Using in vivo mouse metastatic models, based on the intravenous injection of these cells into the tail vein, indicated that MACC1-AS1 overexpression significantly promoted lung metastasis (Fig. 2a). Next, H&E staining demonstrated that the relative area of the metastatic nodules in lung was larger with these cells, suggesting a growth advantage for the MACC1-AS1 overexpression group, though the number of metastatic nodules was not significantly different (Fig. 2b-c). IHC staining showed that MACC1-AS1 promoted MACC1 expression, which was consistent with clinical sample analysis. In addition, staining for the proliferation-related nuclear antigen Ki-67 and oxidative stress marker 8-OHdG was applied to show that MACC1-AS1 promotes tumor cell proliferation and mitigates oxidative stress (Fig. 2b and d). Taken together, these data suggested that MACC1-AS1 played an important role in GC progression.

Since in vivo results indicated a role for MACC1-AS1 in metastasis, we next examined the effects of MACC1-AS1 on migration and invasion. Wound scratch assays and transwell assays indicated that MACC1-AS1 enhanced GC cell migration and invasion capacity (Additional file 6: Figure S3C–E); however, it did not significantly affect the expression of the epithelial-mesenchymal transition (EMT) markers E-cadherin and N-cadherin (Additional file 6: Figure S3F). These results suggested that MACC1-AS1 promoted migration and invasion, but potentially not modulating EMT.

In addition to metastasis, MACC1 is associated with enhanced glycolysis based on PET-CT image analysis in GC patients and in vivo animal models; in addition, metabolic stress can promote MACC1 expression [9]. In the clinic, different therapies can induce metabolic stress, exemplified by chemotherapy agent-induced ROS overload and anti-angiogenic-derived nutrient deprivation [10]. Thus, we utilized non-glucose medium to mimic metabolic stress, and ROS was found to be increased while the ROS scavenger N-Acetyl-L-cysteine (NAC) could reverse the effect (Additional file 7: Figure S4A-B). Further, MACC1-AS1 was upregulated under glucose deprivation (Fig. 2e), and it was also induced by the glycolysis inhibitor 2-deoxyglucose (2-DG) and the oxidative agent H_2_O_2_ in a time-dependent manner (Fig. 2f-g), but this was reversed by the NAC treatment (Fig. 2h). In addition, MACC1-AS1 was also upregulated under culture conditions of sustained low glucose for 1 month (Fig. 2i), and concurrently glucose transporter 1 (GLUT1) and hexokinase 2 (HK2) were elevated (Fig. 2j). Meanwhile, IHC staining of in vivo lung metastatic tumor tissues also showed MACC1-AS1 enhanced the expression of GLUT1, HK2, and LDH (Fig. 2k-l), indicating a potential role for MACC1-AS1 in glucose metabolism and redox state maintenance.

### MACC1-AS1 promotes GC cell viability under metabolic stress

**Fig. 3:**
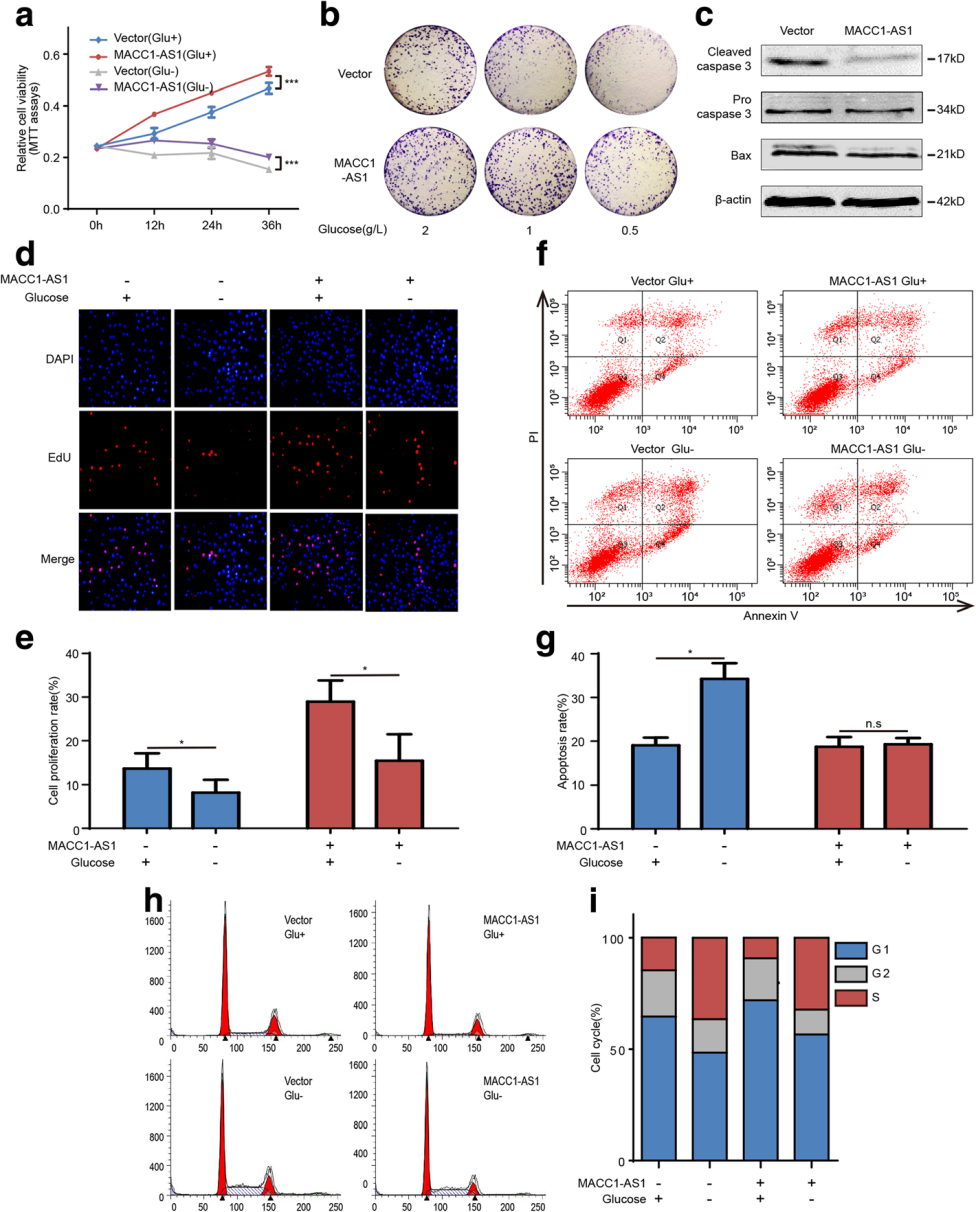
MACC1-AS1 promotes GC cell viability under metabolic stress. **a**-**b** MTT and colony formation assays showing that overexpression of MACC1-AS1 promoted cell viability with glucose deprivation; ***P < 0.001. **c** Western blotting results showing that MACC1-AS1 decreases the expression of bax and cleaved caspase 3 in MKN45. **d**-**e** EdU cell proliferation assays showing that MACC1-AS1 promoted cell viability in MKN45 following 12 h of culture in glucose-free medium; *P < 0.05. **f**-**g** Flow cytometry results showing that MACC1-AS1 inhibited apoptosis induced by glucose deprivation for 12 h in MKN45; *P < 0.05. **h**-**i** Flow cytometry results showing that MACC1-AS1 alleviated glucose deprivation-derived S phrase block

Since MACC1-AS1 was found to be upregulated under metabolic stress, we investigated its effects on GC cell survival in vitro. MACC1-AS1 promoted cell viability either in normal or glucose-deprived conditions, as determined by MTT assays and colony-forming assays (Fig. 3a-b); it also suppressed the expression of caspase 3 and bax, indicating decreased apoptosis (Fig. 3c). 5-ethynyl-2′-deoxyuridine (EdU) cell proliferation assays and flow cytometry further validated that MACC1-AS1 could significantly increase cell survival under conditions of glucose deprivation (Fig. 3d-g). In addition, based on cell cycle analysis, MACC1-AS1 expression mitigated the glucose-deprivation-induced S phase block, which was in consistent with the observed substantial reduction in apoptosis with glucose deprivation (Fig. 3h-i).

### MACC1-AS1 promotes cell survival by enhancing metabolic plasticity

**Fig. 4:**
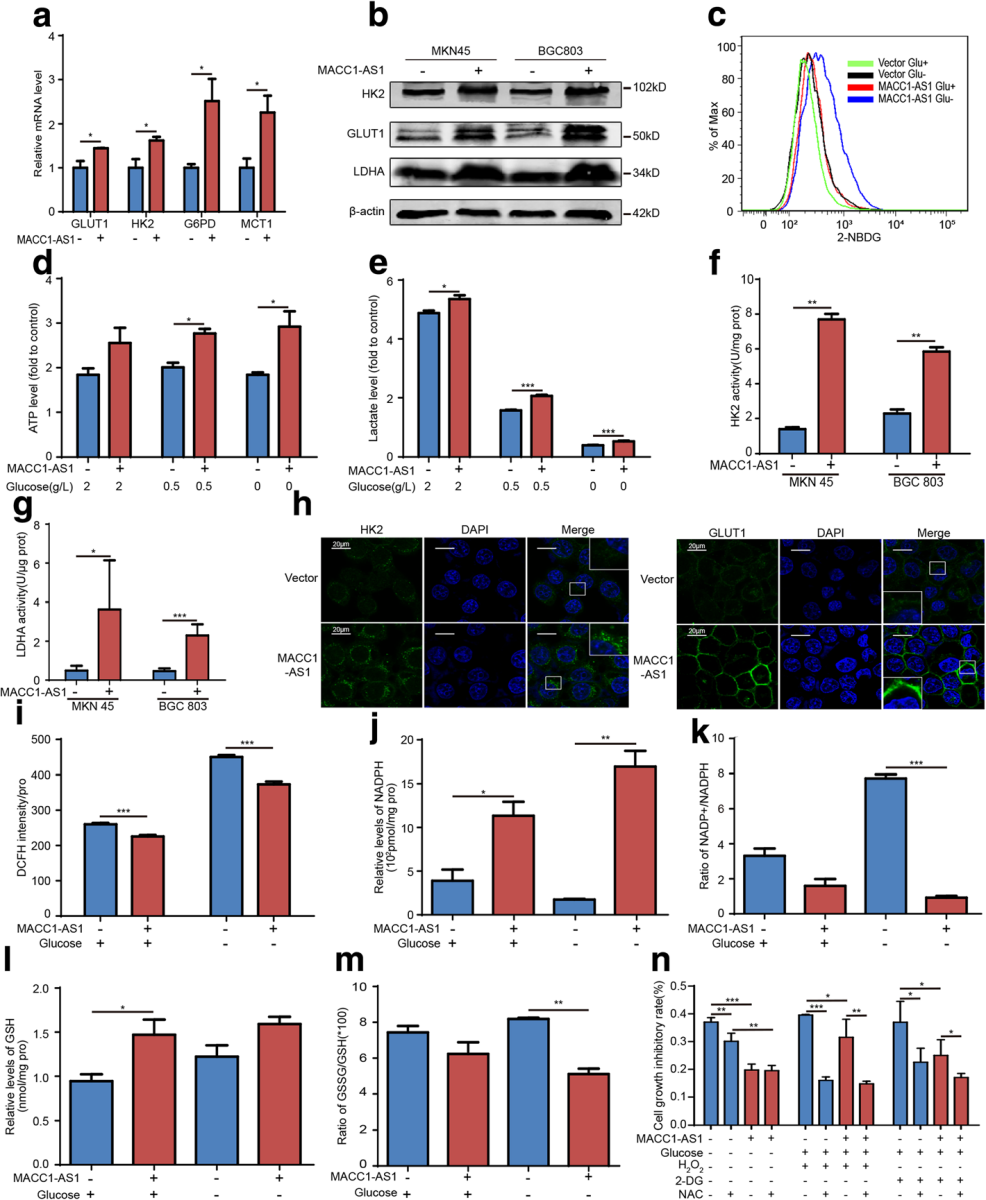
MACC1-AS1 promotes GC cell survival by enhancing metabolic plasticity. **a** qPCR results showing that MACC1-AS1 promoted the expression of GLUT1, HK2, G6PD, and MCT1 at the mRNA level in MKN45; *P < 0.05. **b** Western blotting results showing that MACC1-AS1 upregulated the expression of GLUT1, HK2, and LDH at the protein level in MKN45. **c** 2-NBDG uptake assays showing that MACC1-AS1 promoted glucose absorption either in conditions of normal or deprived glucose. **d**-**e** MACC1-AS1 promoted ATP (D) and lactate (E) production under conditions of glucose deprivation, based on ATP assay kit and lactate assay kit; *P < 0.05, ***P < 0.001. **f**-**g** MACC1-AS1 promoted HK (F) and LDH (G) enzyme activity based on HK and LDH assay kit; *P < 0.05, ***P < 0.001. **h** Immunofluorescence results showing that MACC1-AS1 upregulated GLUT1, HK2 and LDH expression in MKN45. Specifically, GLUT1 was showed to be increased on distribution to the cell membrane, and HK2 to the region surrounding the mitochondria. **i** MACC1-AS1 mitigated reactive oxygen species (ROS) accumulation in MKN45 with glucose deprivation for 12 h, measured by DCFH-DA ROS assay kit; ***P < 0.001. **j**-**k** MACC1-AS1 promoted NADPH production (J) and decreased the NADP+/NADPH ratio with glucose deprivation (K), based on NADP/NADPH quantitation colorimetric kit; *P < 0.05, **P < 0.01, ***P < 0.001. **l**-**m** MACC1-AS1 promoted glutathione (GSH) production (L) and decreased the GSSG/GSH ratio under glucose deprivation (**m**), based on Glutathione (GSH/GSSG/Total) fluorometric assay kit; **P < 0.01. **n** MTT assays showing that MACC1-AS1 promoted cell viability with glucose deprivation, H_2_O_2_, and 2-DG treatment; NAC was used as a positive control to scavenge ROS; *P < 0.05, **P < 0.01, ***P < 0.001

Given that MACC1-AS1 was associated with glucose metabolism and redox state, we further examined their potential regulation. MACC1-AS1 significantly upregulated glycolysis-associated GLUT1, HK2, G6PD, and MCT1 expression at the mRNA level (Fig. 4a), and enhanced GULT1, HK2, and LDH expression at the protein level (Fig. 4b). Further, MACC1-AS1 accelerated glucose uptake based on 2-NBDG assays (Fig. 4c). It also increased ATP and lactate production either in conditions of normal or deprived glucose (Fig. 4d-e); the activity of the key glycolytic enzymes HK2 and LDHA was also increased (Fig. 4f-g). This was consistent with immunofluorescence showing increased distribution of GLUT1 to the cell membrane and HK2 to the region surrounding the mitochondria, indicating enhanced glucose absorption through GLUT1 and stabilization of mitochondrial membrane potential by HK2 binding [11] (Fig. 4h).

Since enhanced glycolysis might facilitate ROS homeostasis by providing components required for antioxidant production, we examined ROS levels and the synthesis of the main cellular antioxidants NADPH and GSH. Results showed that glucose deprivation induced ROS accumulation, whereas MACC1-AS1 partially abrogated this effect (Fig. 4i). MACC1-AS1 significantly increased NADPH and GSH levels, and accordingly, NADP+/NADPH and GSSG/GSH ratios decreased either in conditions of normal or deprived glucose (Fig. 4j-m). MTT assays showed that MACC1-AS1 reversed the inhibition of cell viability caused by glucose deprivation, H_2_O_2_, and 2-DG treatment, and exerted similar effects as the antioxidant NAC (Fig. 4n). These results indicated that MACC1-AS1 enhances glycolysis and antioxidant capacity, facilitating metabolic plasticity and contributing to the cytoprotective effect observed under conditions of metabolic stress.

### MACC1-AS1 promotes metabolic plasticity though MACC1 regulation

**Fig. 5:**
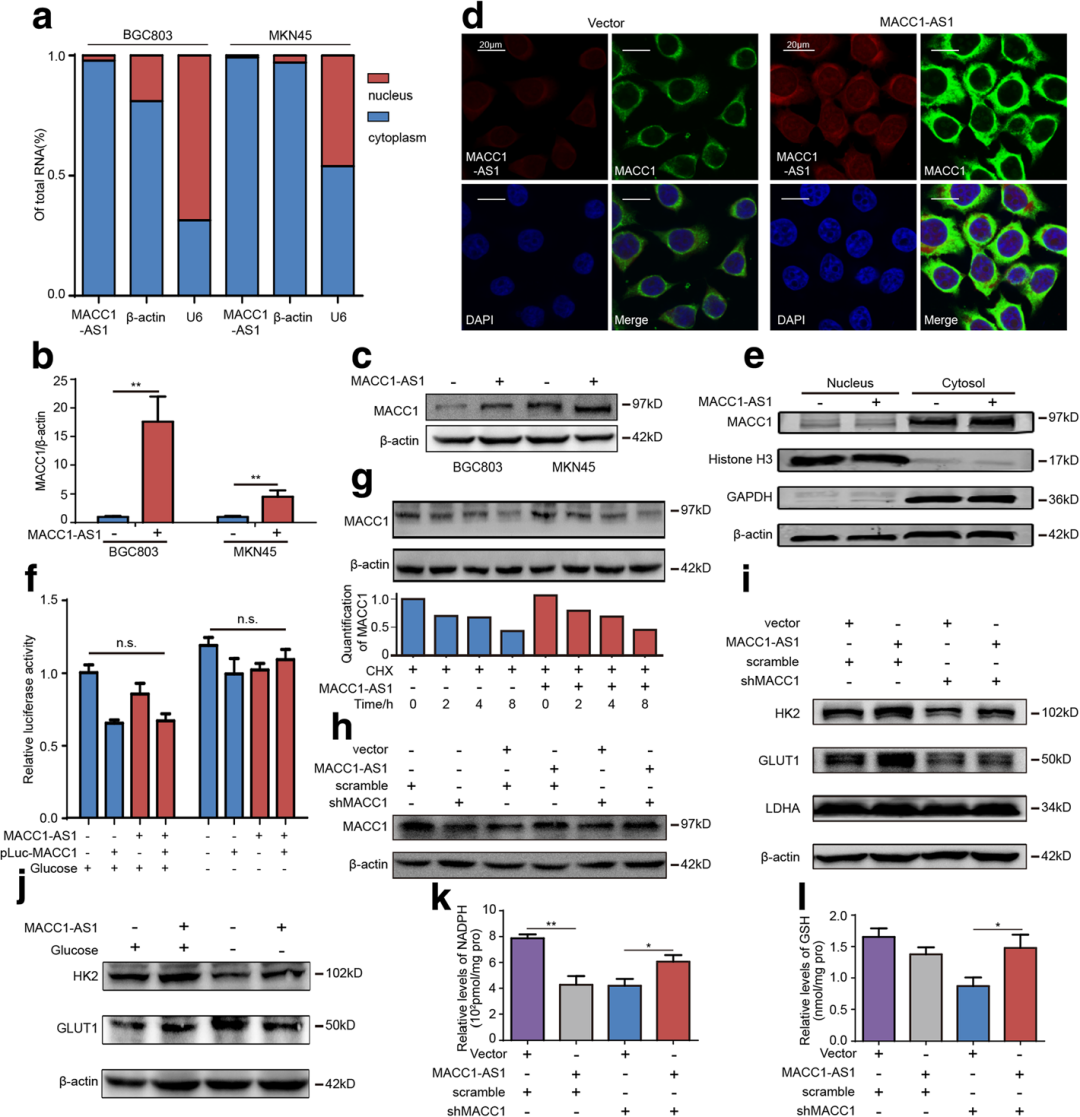
MACC1-AS1 promotes metabolic plasticity in GC cells though MACC1 regulation. **a** qPCR results showing that MACC1-AS1 was located in cytoplasm by fractionation and detection of nuclear and cytoplasmic MACC1-AS1. **b**-**c** qPCR and western blottingresults showing that MACC1-AS1 promoted MACC1 expression at the mRNA and protein level; **P < 0.01. **d** Combined IF/FISH results showing that MACC-AS1 promoted MACC1 expression, while had no influence on the cytoplasm-nuclear distribution of MACC1. **e** Western blotting results showing that MACC1-AS1 did not affect the distribution of MACC1 in the cytoplasm and nucleus. **f** Luciferase assays indicating that MACC1-AS1 did not influence MACC1 promoter transcriptional activity in MKN45 either in conditions of normal or deprived glucose. **g** MACC1-AS1 did not affect protein stability, as assessed by cyclohexamide-induced MACC1 degradation (100 μg/mL), and subsequent western blotting. **h** Decreased MACC1 expression by shRNA interference was reversed by MACC1-AS1 overexpression, based on western blotting. **i** MACC1-AS1 partially reversed the MACC1 shRNA-induced GLUT1, HK2, and LDH suppression based on western blotting. **j** Western blotting results showing that MACC1-AS1 maintained GLUT1 and HK2 expression under glucose deprivation. **k**-**l** NADPH and GSH were decreased after MACC1 silencing, while this effect was abrogated by MACC1-AS1 overexpression

As previously described, both TCGA data and IHC analysis indicated a positive correlation between MACC1-AS1 and MACC1. In addition, given the close genomic proximity of MACC1-AS1 and MACC1, we hypothesized that MACC1-AS1 could exert biologic effects by modulating MACC1. Since the location of lncRNA generally determines function, we performed fluorescence in situ hybridization (FISH) and RNA fractionation of the nucleus and cytoplasm, and identified that MACC1-AS1 mainly resides in the cytoplasm, indicating the potential of post-transcriptional regulation (Fig. 5a).

Next, we performed qPCR and western blotting to examine the effects of MACC1-AS1 on MACC1. The results showed that MACC1 expression was markedly increased, at both the mRNA and protein level, after MACC1-AS1 overexpression (Fig. 5b-c). Further, we performed FISH of MACC1-AS1 followed by immunofluorescent (IF) co-staining of MACC1, which indicated that MACC1-AS1 did not affect the nuclear-cytoplasmic distribution of MACC1 (Fig. 5d-e). In addition, MACC1-AS1 did not transcriptionally control MACC1 based on luciferase assays (Fig. 5f); further, MACC1-AS1 did not affect protein stability, based on experiments in which GC cells were cultured with cycloheximide to inhibit new protein synthesis (Fig. 5g). These results suggested that MACC1-AS1 might regulate MACC1 expression post-transcriptionally.

Further, we explored whether MACC1-AS1 regulates glycolysis through MACC1. Rescue experiments showed that decreased MACC1 expression through shRNA interference could be reversed by MACC1-AS1 overexpression (Fig. 5h). Silencing MACC1 inhibited the expression of GLUT1, HK2, and LDH, but overexpressing MACC1-AS1 partially abrogated this effect (Fig. 5i). Besides, MACC1-AS1 promoted HK2 expression and maintained GLUT1 expression under glucose deprivation (Fig. 5j). Moreover, consistent with declined glycolysis and impaired antioxidant production and elimination, NADPH and GSH were decreased after MACC1 silencing, but it was reversed by MACC1-AS1 overexpression (Fig. 5k-l). These results indicated that MACC1-AS1 is upregulated under metabolic stress, and promoted metabolic plasticity through MACC1 –mediated glycolysis and redox maintenance.

### MACC1-AS1 promotes MACC1 mRNA stability via the AMPK/Lin28 pathway

**Fig. 6:**
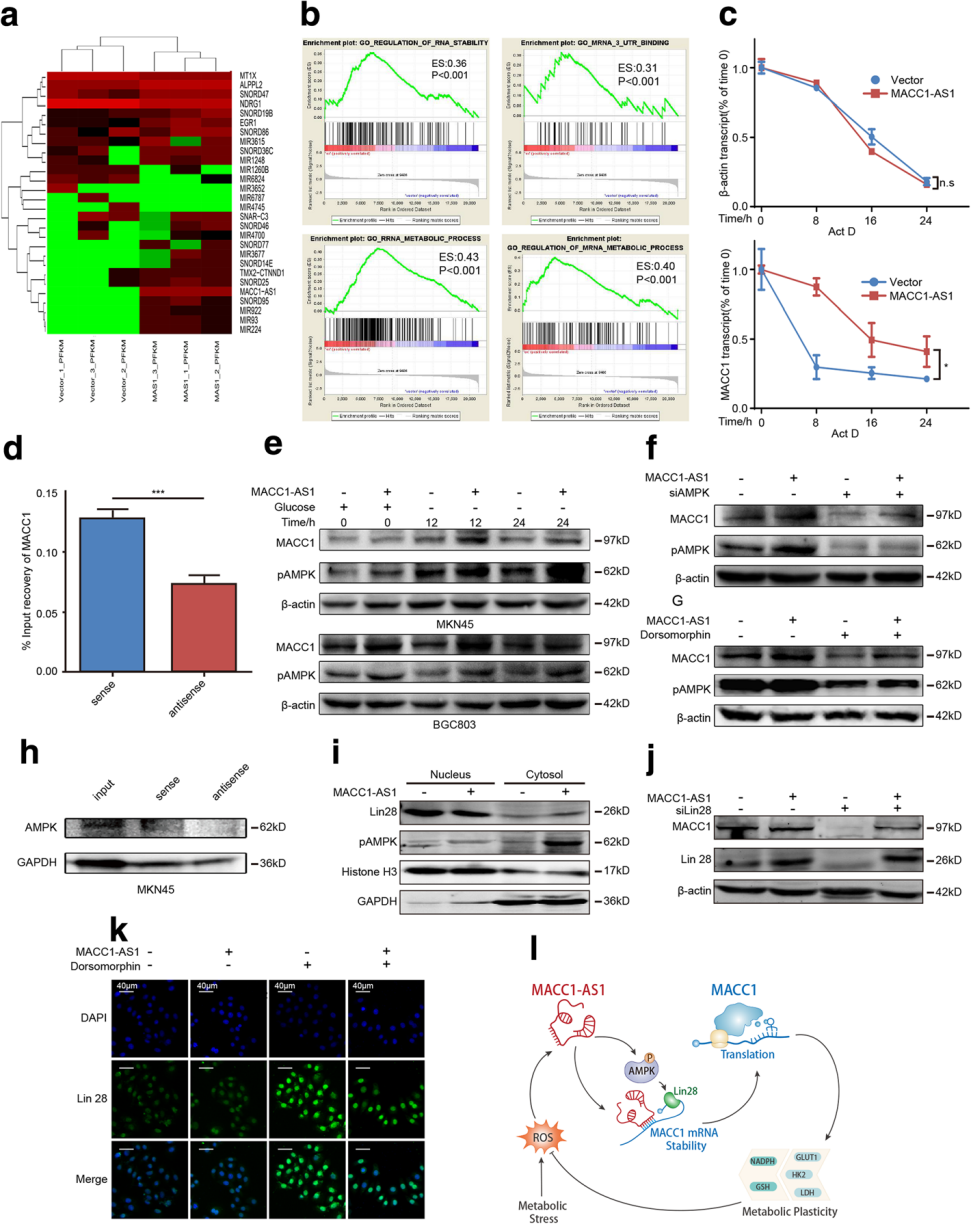
MACC1-AS1 promotes MACC1 mRNA stability via the AMPK/Lin28 pathway in GC. **a** Hierarchical clustering analysis of differentially expressed genes between vector and MACC1-AS1 overexpression groups, based on RNA-seq analysis. **b** GSEA analysis showing that differentially expressed genes with MACC1-AS1 overexpression were enriched in mRNA stability and binding pathways. **c** qPCR results showing that MACC1-AS1 decreased MACC1 mRNA degradation induced by RNA synthesis inhibitor Act D (10 μg/mL); *P < 0.05. **d** Affinity pull-down assays showing that MACC1-AS1 can physically bind to MACC1 mRNA; ***P < 0.001. **e** MACC1-AS1 promoted MACC1 expression concomitantly with AMPK activation, based on western blot analysis. **f**-**g** MACC1 levels were decreased with AMPK silencing (**f**) or dorsomorphin inhibition (50 μM) (**g**), whereas MACC1-AS1 reversed this effect. **h** RNA pull down assays showing that MACC1-AS1 did not interact physically with AMPK in MKN45. **i** MACC1-AS1 promoted cytoplasmic Lin28 distribution, but decreased its nuclear distribution in MKN45; concurrently phosphorylated AMPK was increased in both the cytoplasm and nucleus with MACC1-AS1, based on nuclear fractionation and western blotting. **j** Immunofluorescence showing that AMPK inhibition enhanced nuclear Lin28 distribution, whereas MACC1-AS1 partially abrogated this effect. **k** MACC1-AS1 restored MACC1 expression after Lin28 silencing-induced MACC1 suppression, based on western blot analysis. **l** Schematic representation of the pathway through which glucose deprivation-induced expression of MACC1-AS1 promotes MACC1 mRNA stability and enhances plasticity through glycolysis and antioxidant production

We further suspected that MACC1-AS1 might promote MACC1 mRNA stability. It has been reported that certain antisense lncRNAs might regulate target genes by binding to mRNA [12]. Through bioinformatic analysis, we found that MACC1-AS1 contains a binding site for MACC1 mRNA [13] (http://www.herbbol.org:8001/lrt/) (Additional file 1: Tables S3 and S4). In addition, MACC1-AS1 was mainly located in the cytoplasm, where the regulation of mRNA stability is permitted (Fig. 5a). Moreover, aforementioned transcriptional regulation and modulation of protein stability were previously excluded. Lastly, we performed RNA-seq to profile gene expression after MACC1-AS1 overexpression, and Gene Set Enrichment Analysis (GSEA) analysis identified multiple molecular pathways related to RNA binding, stability, and metabolism (Fig. 6a-b).

Thus, we performed mRNA degradation assays using actinomycin D (Act D) to inhibit de novo mRNA transcription; qPCR analysis showed MACC1-AS1 significantly mitigated the degradation of remaining MACC1 mRNA (Fig. 6c). The physical interaction between MACC1-AS1 and MACC1 was further validated by affinity pull-down of endogenous MACC1 mRNA using in vitro transcribed, biotin-labeled MACC1-AS1 (Fig. 6d). These results showed that MACC1-AS1 promotes mRNA stability through direct binding.

AMPK plays important roles in modulating mRNA stability, especially under conditions of stress [5]. Since MACC1 was upregulated by AMPK, we further investigated the association between this kinase and MACC1-AS1. Interestingly, we found that MACC1-AS1 promoted AMPK phosphorylation concurrent with MACC1 elevation under conditions of glucose deprivation (Fig. 6e), suggesting it might modulate MACC1 expression in an AMPK-dependent manner. Using the AMPK inhibitor dorsomorphin and siRNA interference, MACC1-AS1 was shown to abrogate the AMPK inhibition-induced suppression of MACC1 (Fig. 6f-g). However, RNA pull-down assays with subsequent mass spectrometry did not uncover physical binding between MACC1-AS1 and AMPK (Fig. 6h and Additional file 8: Table S5).

To further identify the role of AMPK in MACC1 regulation by MACC1-AS1, we assessed RNA binding proteins (RBPs), which play significant roles in the regulation of mRNA stability. It was reported that AMPK is essential for promoting mRNA stability through the modulation of RBP. Among potential RBPs, Lin28 was found to interact with MACC1 mRNA through crosslinking immunoprecipitation and high-throughput sequencing (CLIP-seq) [14] (Additional file 1: Table S6), suggesting a potential role for MACC1 mRNA stability. Through immunofluorescence, we found that Lin28 was mainly located in the nucleus in MKN45 cells (Fig. 6k). Since Lin28 exerted its mRNA-binding role mainly in the cytoplasm, we examined the effects of MACC1-AS1 on Lin28 distribution. The results showed that overexpression of MACC1-AS1 promoted Lin28 cytoplasmic distribution, but suppressed nuclear distribution. Concomitantly, phosphorylated AMPK was increased both in the cytoplasm and nucleus, suggesting that AMPK is involved in Lin28 translocation after MACC1-AS1 overexpression (Fig. 6i). Accordingly, we inhibited AMPK using dorsomorphin, and the results showed a dramatic accumulation of Lin28 in the nucleus, whereas MACC1-AS1 partially abrogated this effect (Fig. 6j). Further rescue assays also indicated that silencing Lin28 significantly reduced MACC1 expression, whereas its expression was restored with MACC1-AS1 overexpression (Fig. 6k). Taken together, these results demonstrated that MACC1-AS1 promotes MACC1 mRNA stability and expression through AMPK activation and subsequent Lin28 translocation from the nucleus to the cytoplasm.

## Discussion

We previously reported that MACC1 is overexpressed in GC and associated with GC metastasis, chemoresistance, and poor prognosis [15, 16]. Our present study identifies that MACC1-AS1 is highly elevated and exhibits a robust correlation with MACC1 expression in GC. In addition, high expression of MACC1-AS1 was associated with poor prognosis, suggesting it could be a biomarker to identify patients at a higher risk of GC progression. Mechanistically, MACC1-AS1 regulates MACC1 expression and promotes metabolic plasticity by enhancing glycolysis and antioxidant capacity, conferring a proliferation advantage under metabolic stress. Specifically, MACC1-AS1 was shown to promote MACC1 mRNA stability through physical binding to MACC1 mRNA, and that was shown to be concomitantly coordinated by AMPK activation and subsequent Lin28 translocation (Fig. 6l).

MACC1 functions as a key regulator of the HGF/c-MET axis, which is a promising target for cancer therapy [17]. However, the results of clinical trials targeting c-MET have failed to achieve better prognosis for GC patients [18]. The reason for this is unclear, and understanding a c-MET-associated regulator might help us to better explore more effective ways to target HGF/c-MET. In our study, the MACC1-AS1/MACC1 axis functioned as a modulator of metabolic plasticity by promoting glycolysis and antioxidant production. The HGF/c-MET has been reported to drive metabolic adaptation to prevent glucose deprivation-induced apoptosis [19]. In addition, inhibition of this pathway perturbs redox homeostasis by downregulating NADPH production [20]; thus we suggest that inhibition of MACC1-AS1/MACC1 might result in synergetic anti-tumor effects, when combined with the clinical application of HGF/c-MET inhibitors, by targeting metabolic plasticity.

Extensive research has indicated that metabolic plasticity is essential for cancer cells to survive hostile nutrient-deprived environments [21–23]. Cancer cells might select for highly metabolically plastic cells to survive. A recent study indicated that hypoglycemic tumor microenvironment increases resistance to cytotoxic agents in GC, especially in tumor cells with an enhanced glycolysis phenotype [24]; glucose deprivation was also shown to drive the acquisition of KRAS mutations in colorectal cancer, which mediates tumor plasticity in multiple ways [25–27]. Glucose deprivation could induce a mesenchymal phenotype through metabolic plasticity to overcome apoptosis during metastatic colonization and the acquisition of chemotherapy resistance [10, 28–30]. Therefore, targeting metabolic plasticity could affect tumor growth, metastasis, and treatment efficacy [2, 31]. Our current study indicated that MACC1-AS1 is a stress-responsive gene, as it was shown to promote metabolic adaptation by upregulating GLUT1, HK2, and LDHA expression. Consistent with the fact that MACC1-AS1 predicts poor survival, the stress-responsive genes implicated in our study, including AMPK, Lin28, MACC1, and GLUT1, also predict poor outcomes in GC (Additional file 9: Figure S5A–D). These results indicate that such genes might promote plasticity by modulating tumor metabolism, highlighting the association between MACC1-AS1 and metabolic plasticity in GC and suggesting a new strategy for GC treatment.

The mRNA decay and stability response under metabolic stress is essential for mediating tumor plasticity. Compared to the lag time required to produce proteins de novo, modulating mRNA stability is a fast-acting strategy that cancer cells could utilize to for rapid adaptation and maximum cell survival [32]. AMPK is important for this process through its ability to regulate mRNA translation, elongation, and stability [33]. It was reported that AMPK promotes the mRNA stability of VEGF and COX2 by modulating RBPs [34, 35]. Lin28 is also an evolutionarily-conserved RBP. Although it is particularly known to regulate the Lin28/let-7 oncogenic pathway, its role in mRNA stability cannot be ignored. Lin28 can physically bind bulk mRNAs, many of which have been implicated in the regulation of metabolic processes, such as IGF2, IMP, and OCT4. It is also a component of stress granules where mRNAs are sequestered in RNA-protein complexes to adapt to conditions of stress [36, 37]. However, whether AMPK is involved in the function of Lin28 has not been reported. In our study, MACC1-AS1 promoted AMPK activation and subsequent Lin28 translocation, which indicated that AMPK requires Lin28 subcellular trafficking to exert its roles in the maintenance of RNA stability. In addition, our present study demonstrated that MACC1-AS1 can physically bind MACC1 mRNA, forming RNA-RNA complexes, to stabilize MACC1 mRNA [38]. However, whether MACC1-AS1 associates with Lin28 and mediates its recruitment to MACC1 mRNA requires further investigation.

## Conclusion

In conclusion, we determined that MACC1-AS1 was highly expressed in GC tissues, which was closely associated with clinical stage and survival outcomes in GC patients. Furthermore, the effects of MACC1-AS1 on GC cell proliferation and metastasis indicated that MACC1-AS1 could promote GC tumorigenesis both in vitro and in vivo. Specifically, MACC1-AS1 was upregulated under metabolic stress and facilitated metabolic plasticity by promoting MACC1 expression through mRNA stabilization. This study may provide a potential novel therapeutic target for the diagnosis and treatment of GC.

## Additional files

Additional file 1: **Table S3.** Prediction of binding site that MACC1-AS1 interacts with MACC1 mRNA. **Table S4.**. Negative binding potential of MACC1-AS1 with reference gene. **Table S6.** Potential RNA binding protein interacting with MACC1 mRNA. **Table S7.** Primer sequence. (DOCX 6123 kb)

Additional file 2: **Figure S1.** MACC1-AS1 is a lncRNA elevated in GC with no coding potential. (TIFF 360 kb)

Additional file 3: **Table S1.** Analysis of upreguated differential genes in STAD from TCGA. (XLSX 447 kb)

Additional file 4: **Table S2.** Analysis of genes correlated with MACC1 in STAD from TCGA. (XLSX 14019 kb)

Additional file 5: **Figure S2.** MACC1-AS1 is correlated with MACC1 expression. (TIFF 146 kb)

Additional file 6: **Figure S3.** MACC1-AS1 promotes migration and invasion in vitro*. (TIFF 4128 kb)*

Additional file 7: **Figure S4.** ROS is induced by glucose deprivation. (TIFF 251 kb)

Additional file 8: **Table S5.** Mass spectrometry analysis of potential proteins binding with MACC1-AS1 followed by RNA pull down assays. (XLSX 58 kb)

Additional file 9: **Figure S5.** MACC1-AS1 associated stress responsive genes are correlated with poor survival. (TIFF 415 kb)

## References

[1] Van Cutsem E, Sagaert X, Topal B, Haustermans K, Prenen H (2016). Gastric cancer. Lancet.

[2] Lehuédé C, Dupuy F, Rabinovitch R, Jones RG, Siegel PM (2016). Metabolic plasticity as a determinant of tumor growth and metastasis. Cancer Res.

[3] Wu Z-Z, Chen L-S, Zhou R, Bin J-P, Liao Y-L, Liao W-J (2016). Metastasis-associated in colon cancer-1 in gastric cancer: beyond metastasis. World J Gastroenterol.

[4] Stylianopoulos T, Martin JD, Snuderl M, Mpekris F, Jain SR, Jain RK (2013). Coevolution of solid stress and interstitial fluid pressure in tumors during progression: implications for vascular collapse. Cancer Res.

[5] Zhao Y, Hu X, Liu Y, Dong S, Wen Z, He W, Zhang S, Huang Q, Shi M (2017). ROS signaling under metabolic stress: cross-talk between AMPK and AKT pathway. Mol Cancer.

[6] Yang F, Xue X, Zheng L, Bi J, Zhou Y, Zhi K, Gu Y, Fang G (2014). Long non-coding RNA GHET1 promotes gastric carcinoma cell proliferation by increasing c-Myc mRNA stability. FEBS J.

[7] Washington K (2010). Of the AJCC cancer staging manual: stomach. Ann Surg Oncol.

[8] Guo Y, Sheng Q, Li J, Ye F, Samuels DC, Shyr Y (2013). Large scale comparison of gene expression levels by microarrays and RNAseq using TCGA data. PLoS One.

[9] Lin L, Huang H, Liao W, Ma H, Liu J, Wang L, Huang N, Liao Y (2015). MACC1 supports human gastric cancer growth under metabolic stress by enhancing the Warburg effect. Oncogene.

[10] Kanska J, Aspuria P-JP, Taylor-Harding B, Spurka L, Funari V, Orsulic S, Karlan BY, Wiedemeyer WR (2017). Glucose deprivation elicits phenotypic plasticity via ZEB1-mediated expression of NNMT. Oncotarget.

[11] Cheung EC, Ludwig RL, Vousden KH (2012). Mitochondrial localization of TIGAR under hypoxia stimulates HK2 and lowers ROS and cell death. Proc Natl Acad Sci.

[12] Huang B, Song J, Cheng Y, Abraham J, Ibrahim S, Sun Z, Ke X, Meltzer S (2016). Long non-coding antisense RNA KRT7-AS is activated in gastric cancers and supports cancer cell progression by increasing KRT7 expression. Oncogene.

[13] Hu R, Sun X (2016). lncRNATargets: a platform for lncRNA target prediction based on nucleic acid thermodynamics. J Bioinform Comput Biol.

[14] Li J-H, Liu S, Zhou H, Qu L-H, Yang J-H (2013). starBase v2. 0: decoding miRNA-ceRNA, miRNA-ncRNA and protein–RNA interaction networks from large-scale CLIP-Seq data. Nucleic Acids Res.

[15] Yang T, He W, Cui F, Xia J, Zhou R, Wu Z, Zhao Y, Shi M (2016). MACC1 mediates acetylcholine-induced invasion and migration by human gastric cancer cells. Oncotarget.

[16] Wang C, Wen Z, Xie J, Zhao Y, Zhao L, Zhang S, Liu Y, Xue Y, Shi M (2017). MACC1 mediates chemotherapy sensitivity of 5-FU and cisplatin via regulating MCT1 expression in gastric cancer. Biochem Biophys Res Commun.

[17] Stein U, Walther W, Arlt F, Schwabe H, Smith J, Fichtner I, Birchmeier W, Schlag PM (2009). MACC1, a newly identified key regulator of HGF-MET signaling, predicts colon cancer metastasis. Nat Med.

[18] Bradley CA, Salto-Tellez M, Laurent-Puig P, Bardelli A, Rolfo C, Tabernero J, Khawaja HA, Lawler M, Johnston PG, Van Schaeybroeck S (2017). Targeting c-MET in gastrointestinal tumours: rationale, opportunities and challenges. Nat Rev Clin Oncol.

[19] Mira A, Morello V, Céspedes MV, Perera T, Comoglio PM, Mangues R, Michieli P (2017). Stroma-derived HGF drives metabolic adaptation of colorectal cancer to angiogenesis inhibitors. Oncotarget.

[20] Lui VWY, Wong EYL, Ho K, Ng PKS, Lau CPY, Tsui SKW, Tsang C-M, Tsao S-W, Cheng SH, Ng MHL (2011). Inhibition of c-met downregulates TIGAR expression and reduces NADPH production leading to cell death. Oncogene.

[21] Ma L, Tao Y, Duran A, Llado V, Galvez A, Barger JF, Castilla EA, Chen J, Yajima T, Porollo A (2013). Control of nutrient stress-induced metabolic reprogramming by PKCζ in tumorigenesis. Cell.

[22] Piskounova E, Agathocleous M, Murphy MM, Hu Z, Huddlestun SE, Zhao Z, Leitch AM, Johnson TM, DeBerardinis RJ, Morrison SJ (2015). Oxidative stress inhibits distant metastasis by human melanoma cells. Nature.

[23] Schafer ZT, Grassian AR, Song L, Jiang Z, Gerhart-Hines Z, Irie HY, Gao S, Puigserver P, Brugge JS (2009). Antioxidant and oncogene rescue of metabolic defects caused by loss of matrix attachment. Nature.

[24] Bhattacharya B, Low S, Soh C, Kamal Mustapa N, Beloueche-Babari M, Koh K, Loh J, Soong R (2014). Increased drug resistance is associated with reduced glucose levels and an enhanced glycolysis phenotype. Br J Pharmacol.

[25] Yun J, Rago C, Cheong I, Pagliarini R, Angenendt P, Rajagopalan H, Schmidt K, Willson JK, Markowitz S, Zhou S (2009). Glucose deprivation contributes to the development of KRAS pathway mutations in tumor cells. Science.

[26] Grabocka E, Bar-Sagi D (2016). Mutant KRAS enhances tumor cell fitness by upregulating stress granules. Cell.

[27] Ying H, Kimmelman AC, Lyssiotis CA, Hua S, Chu GC, Fletcher-Sananikone E, Locasale JW, Son J, Zhang H, Coloff JL (2012). Oncogenic Kras maintains pancreatic tumors through regulation of anabolic glucose metabolism. Cell.

[28] Liu L, Duclos G, Sun B, Lee J, Wu A, Kam Y, Sontag ED, Stone HA, Sturm JC, Gatenby RA (2013). Minimization of thermodynamic costs in cancer cell invasion. Proc Natl Acad Sci.

[29] Simões RV, Serganova IS, Kruchevsky N, Leftin A, Shestov AA, Thaler HT, Sukenick G, Locasale JW, Blasberg RG, Koutcher JA (2015). Metabolic plasticity of metastatic breast cancer cells: adaptation to changes in the microenvironment. Neoplasia.

[30] Viswanathan VS, Ryan MJ, Dhruv HD, Gill S, Eichhoff OM, Seashore-Ludlow B, Kaffenberger SD, Eaton JK, Shimada K, Aguirre AJ (2017). Dependency of a therapy-resistant state of cancer cells on a lipid peroxidase pathway. Nature.

[31] Oizel K, Chauvin C, Oliver L, Gratas C, Geraldo F, Jarry U, Scotet E, Rabe M, Alves-Guerra M-C, Teusan R (2017). Efficient mitochondrial glutamine targeting prevails over glioblastoma metabolic plasticity. Clin Cancer Res.

[32] De Nadal E, Ammerer G, Posas F (2011). Controlling gene expression in response to stress. Nat Rev Genet.

[33] Senft D, Ze'ev AR (2016). Adaptive stress responses during tumor metastasis and dormancy. Trends in cancer.

[34] Yun H, Lee M, Kim S-S, Ha J (2005). Glucose deprivation increases mRNA stability of vascular endothelial growth factor through activation of AMP-activated protein kinase in DU145 prostate carcinoma. J Biol Chem.

[35] Zhang J, Bowden GT (2008). UVB irradiation regulates Cox-2 mRNA stability through AMPK and HuR in human keratinocytes. Mol Carcinog.

[36] Qiu C, Ma Y, Wang J, Peng S, Huang Y (2009). Lin28-mediated post-transcriptional regulation of Oct4 expression in human embryonic stem cells. Nucleic Acids Res.

[37] Shyh-Chang N, Daley GQ (2013). Lin28: primal regulator of growth and metabolism in stem cells. Cell Stem Cell.

[38] Yoon J-H, Abdelmohsen K, Gorospe M (2013). Posttranscriptional gene regulation by long noncoding RNA. J Mol Biol.

