# Supplementary figures and images for: The lncRNA MACC1-AS1 promotes gastric cancer cell metabolic plasticity via AMPK/Lin28 mediated mRNA stability of MACC1
Source: Mol Cancer. 2018 Mar 6;17:69. doi: 10.1186/s12943-018-0820-2 (PMC5838949; doi:10.1186/s12943-018-0820-2)

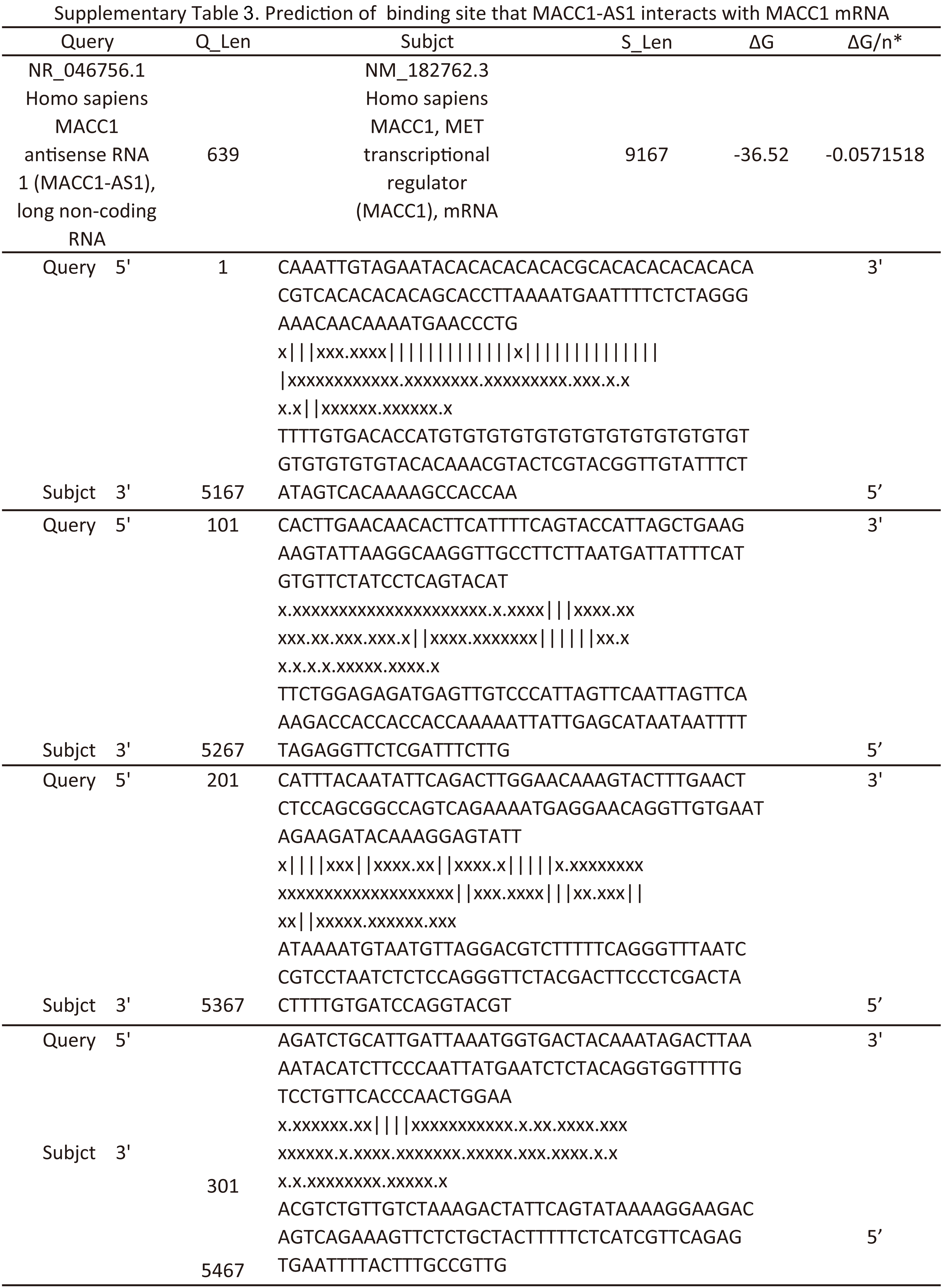


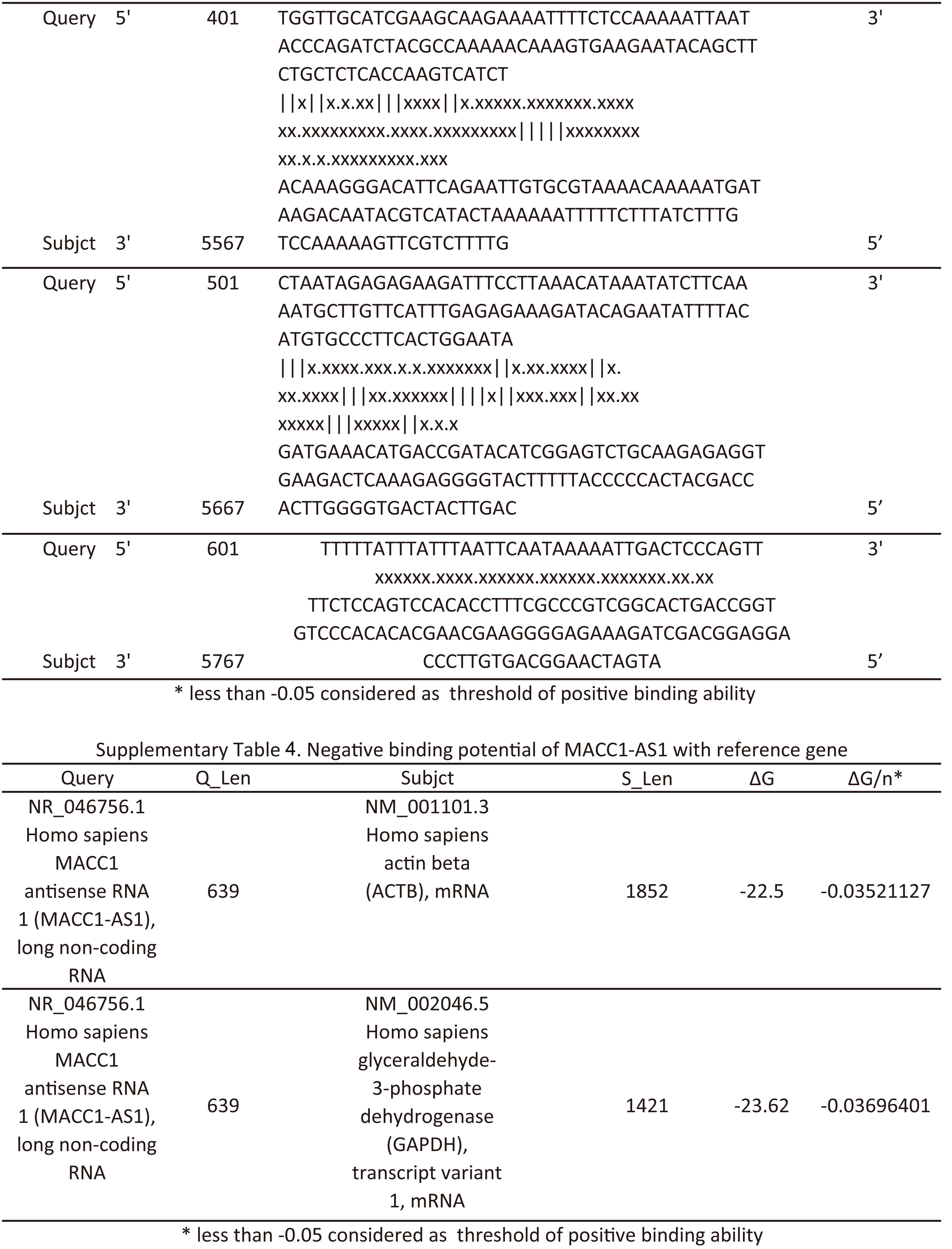


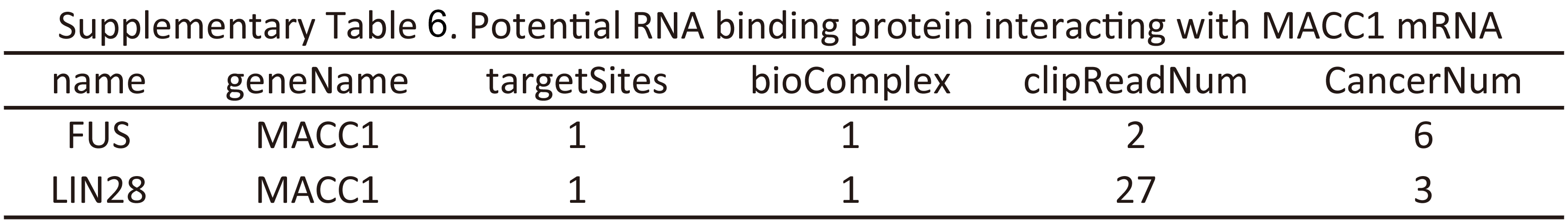
 Data from STARBASE 2.0 database (http://starbase.sysu.edu.cn/index.php)


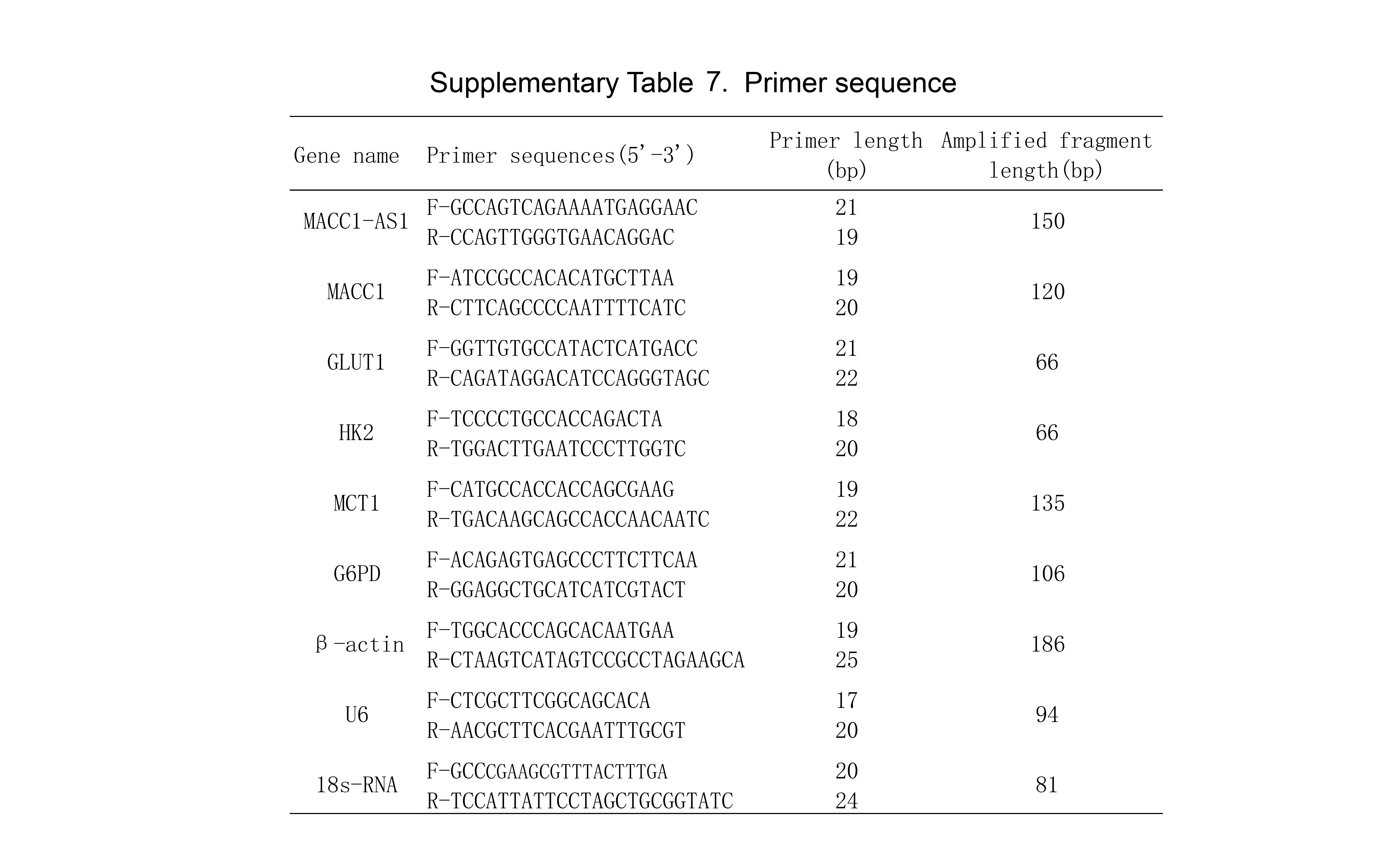

Supplement: Supplementary file 1 — Table S3. Prediction of binding site that MACC1-AS1 interacts with MACC1 mRNA. Table S4.. Negative binding potential of MACC1-AS1 with reference gene. Table S6. Potential RNA binding protein interacting with MACC1 mRNA. Table S7. Primer sequence. (DOCX 6123 kb) [file 12943_2018_820_MOESM1_ESM.docx]

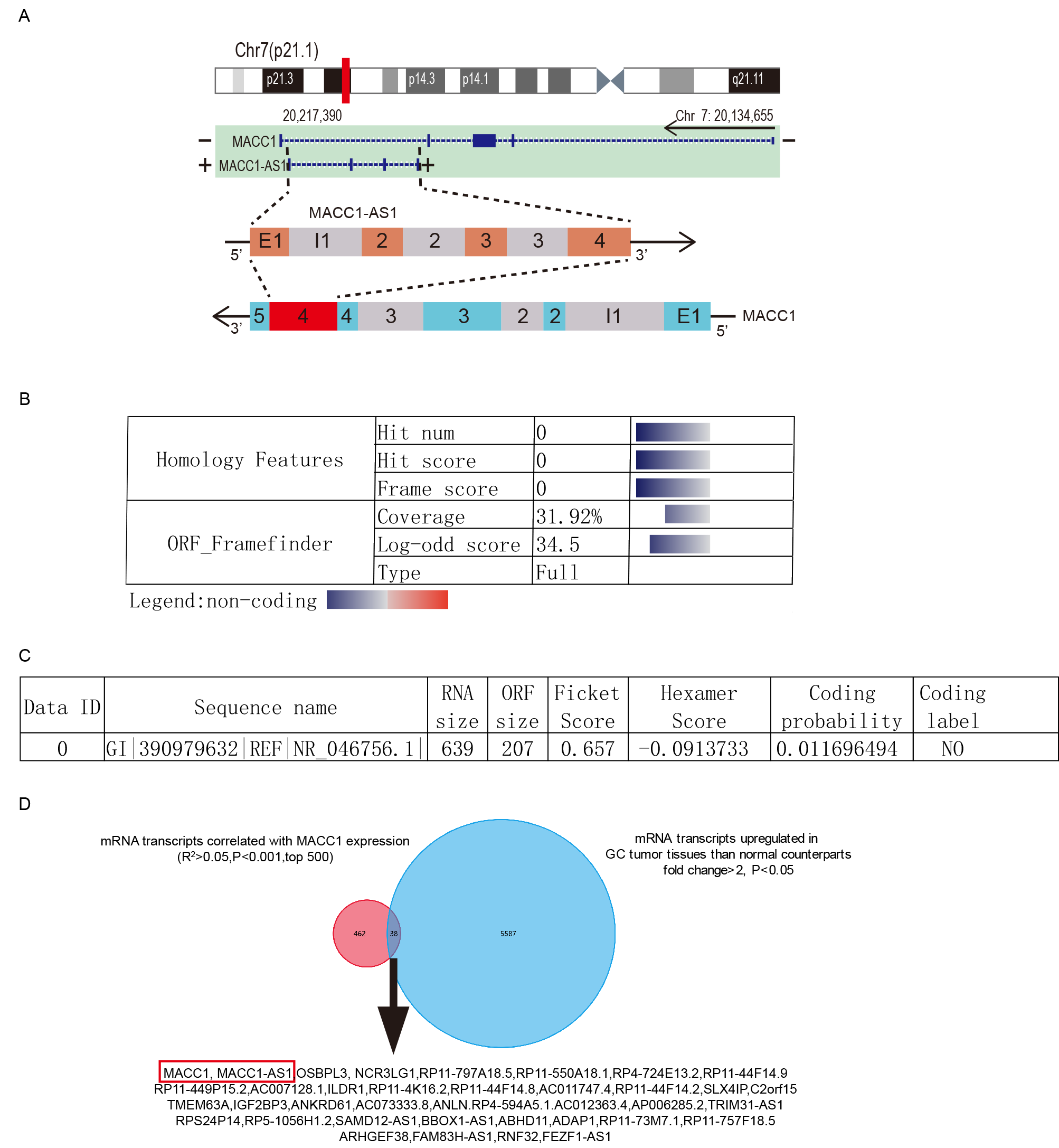

Supplement: Supplementary file 2 — Figure S1. MACC1-AS1 is a lncRNA elevated in GC with no coding potential. (TIFF 360 kb) [file 12943_2018_820_MOESM2_ESM.tif]

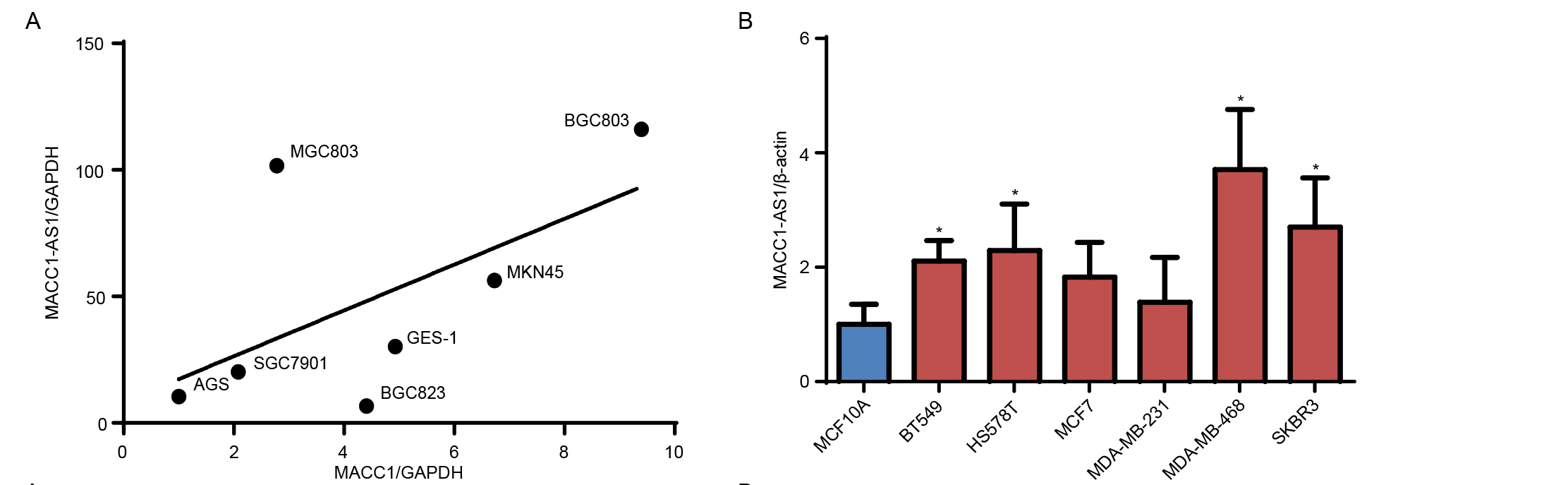

Supplement: Supplementary file 5 — Figure S2. MACC1-AS1 is correlated with MACC1 expression. (TIFF 146 kb) [file 12943_2018_820_MOESM5_ESM.tif]

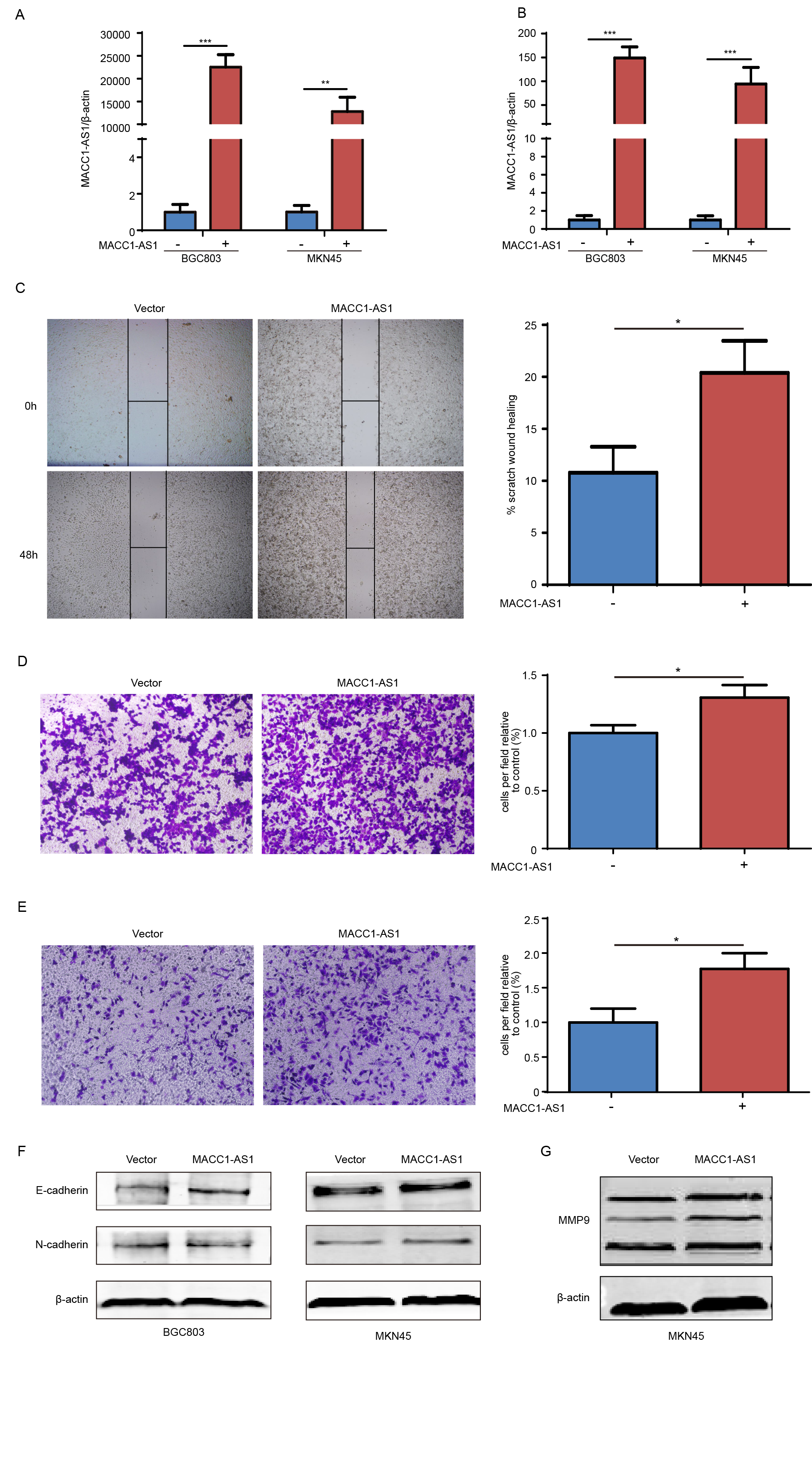

Supplement: Supplementary file 6 — Figure S3. MACC1-AS1 promotes migration and invasion in vitro. (TIFF 4128 kb) [file 12943_2018_820_MOESM6_ESM.tif]

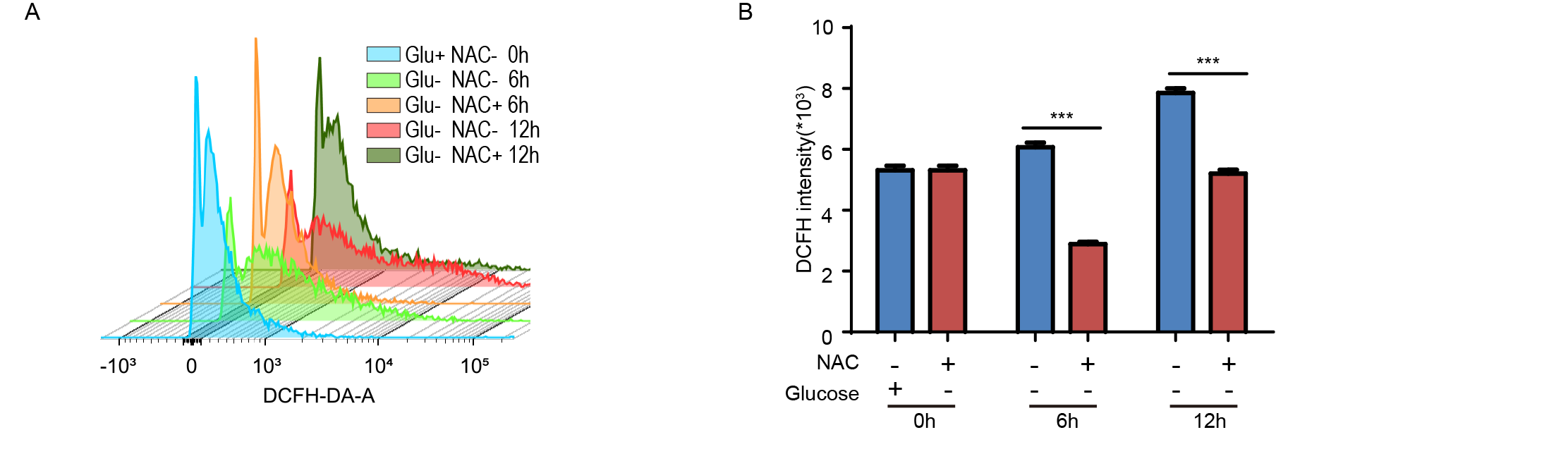

Supplement: Supplementary file 7 — Figure S4. ROS is induced by glucose deprivation. (TIFF 251 kb) [file 12943_2018_820_MOESM7_ESM.tif]

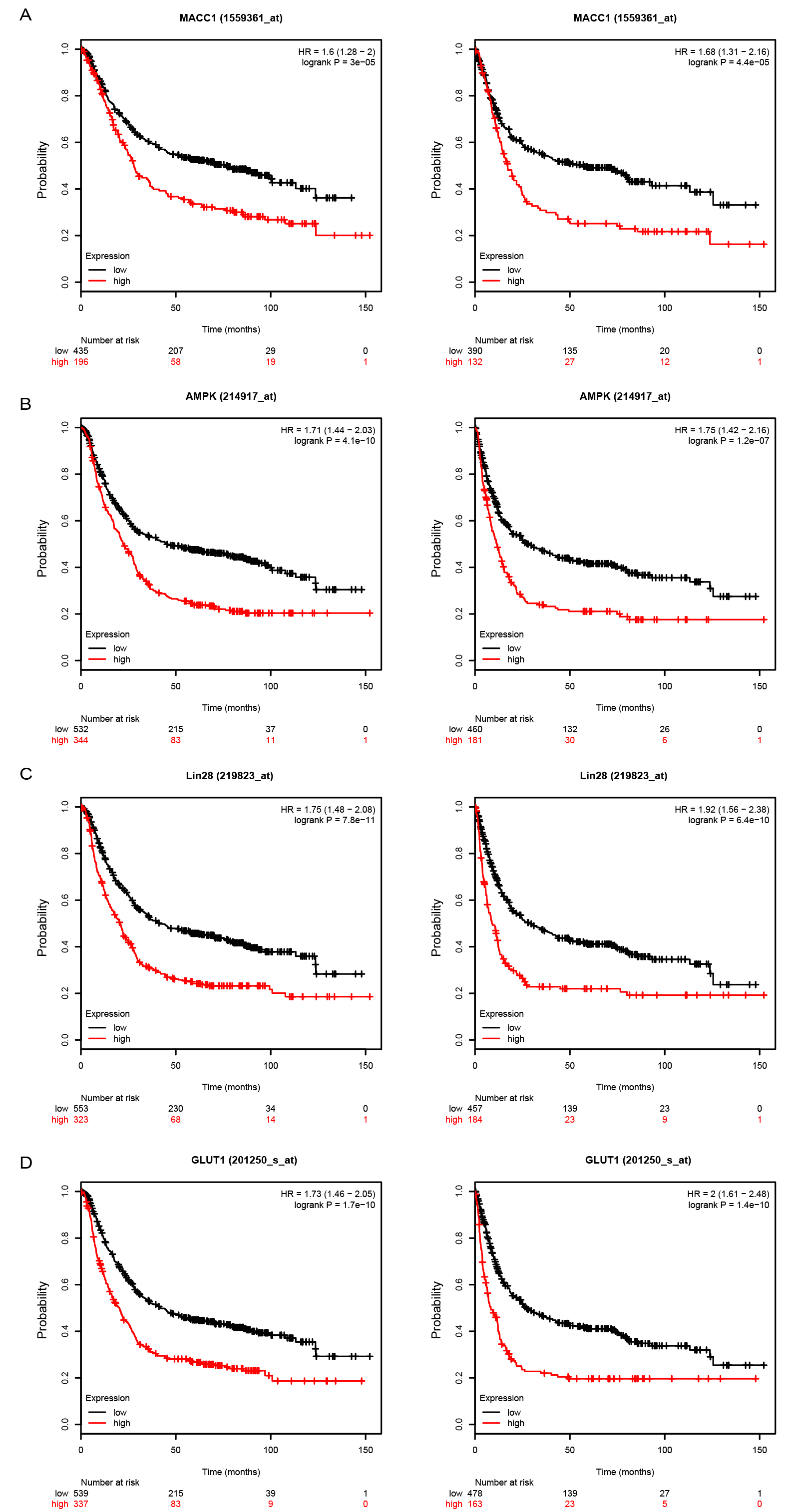

Supplement: Supplementary file 9 — Figure S5. MACC1-AS1 associated stress responsive genes are correlated with poor survival. (TIFF 415 kb) [file 12943_2018_820_MOESM9_ESM.tif]
